# Dynamic Assembly of a Membrane Signaling Complex Enables Selective Activation of NFAT by Orai1

**DOI:** 10.1016/j.cub.2014.04.046

**Published:** 2014-06-16

**Authors:** Pulak Kar, Krishna Samanta, Holger Kramer, Otto Morris, Daniel Bakowski, Anant B. Parekh

**Affiliations:** 1 Department of Physiology, Anatomy, and Genetics, Oxford University, Parks Road, Oxford OX1 3PT, UK

## Abstract

NFAT-dependent gene expression is essential for the development and function of the nervous, immune, and cardiovascular systems and kidney, bone, and skeletal muscle [1]. Most NFAT protein resides in the cytoplasm because of extensive phosphorylation, which masks a nuclear localization sequence. Dephosphorylation by the Ca^2+^-calmodulin-activated protein phosphatase calcineurin triggers NFAT migration into the nucleus [2, 3]. In some cell types, NFAT can be activated by Ca^2+^ nanodomains near open store-operated Orai1 and voltage-gated Ca^2+^ channels in the plasma membrane [4, 5]. How local Ca^2+^ near Orai1 is detected and whether other Orai channels utilize a similar mechanism remain unclear. Here, we report that the paralog Orai3 fails to activate NFAT. Orai1 is effective in activating gene expression via Ca^2+^ nanodomains because it participates in a membrane-delimited signaling complex that forms after store depletion and brings calcineurin, via the scaffolding protein AKAP79, to calmodulin tethered to Orai1. By contrast, Orai3 interacts less well with AKAP79 after store depletion, rendering it ineffective in activating NFAT. A channel chimera of Orai3 with the N terminus of Orai1 was able to couple local Ca^2+^ entry to NFAT activation, identifying the N-terminal domain of Orai1 as central to Ca^2+^ nanodomain-transcription coupling. The formation of a store-dependent signaling complex at the plasma membrane provides for selective activation of a fundamental downstream response by Orai1.

## Highlights

• Ca^2+^ store depletion leads to the formation of a plasmalemmal signaling complex
• AKAP79, with bound calcineurin and NFAT1, couples to the N terminus of Orai1 channels
• Ca^2+^ entry though the channels releases activated NFAT, leading to gene expression
• These results identify a mechanism for selective activation of a response by Orai1

## Teaser

Kar et al. describe the formation of a plasma membrane signaling complex that enables Ca^2+^ microdomains near Orai1 channels to activate nuclear gene expression through the transcription factor NFAT. The complex forms after store depletion and brings calcineurin and NFAT, via the scaffolding protein AKAP79, to the Orai1 Ca^2+^ channels.

## Results and Discussion

Store-operated Ca^2+^ channels are a major conduit for Ca^2+^ influx in nonexcitable cells [6, 7]. The best characterized and most widely distributed store-operated channel is the Ca^2+^ release-activated Ca^2+^ (CRAC) channel [8, 9]. CRAC channels are activated following the loss of Ca^2+^ from the ER. The molecular basis of this process is becoming clear: store depletion leads to oligomerization of the ER protein STIM1, and the oligomers then migrate to ER-plasma membrane junctions, where they bind to the pore-forming subunit of the CRAC channel Orai1 and trigger the channel to open [10]. The crystal structure of *Drosophila* Orai1 reveals the channel to be a hexamer [11].

Local Ca^2+^ signals in the vicinity of open CRAC channels activate important cytoplasmic signaling molecules, including enzymes [12, 13], ion channels [14], and vesicular fusion proteins [15]. In all of these cases, the Ca^2+^-dependent target is closely apposed to the Ca^2+^ channel, rapidly transducing the local Ca^2+^ signal into a physiological output. A more complex scenario arises when the activated target is located at a distance well beyond the realm of the CRAC channel Ca^2+^ microdomain, typically <10–20 nm in spatial extent [16]. This spatial disconnect is seen with certain cytoplasmic enzymes [17] and intracellular transcription factors, including c-*fos* [18, 19] and NFAT [20]. The majority of NFAT protein is retained within the cytoplasm through extensive phosphorylation [1], and dephosphorylation by calcineurin results in migration of the transcription factor into the nucleus. An important but unresolved question is how Ca^2+^ nanodomains near CRAC channels are sensed and relayed to cytoplasmic targets such as NFAT.

**Figure 1:**
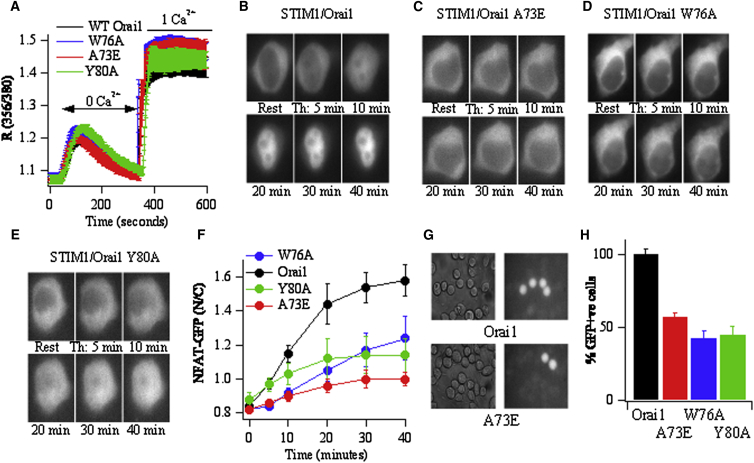
Mutations in the Calmodulin-Binding Domain of Orai1 Impair NFAT Activation (A) Ca^2+^ release and subsequent store-operated Ca^2+^ entry to thapsigargin (2 μM) are shown in HEK293 cells transfected with STIM1 together with either nonmutated (WT) Orai1, A73E Orai1, W76A Orai1, or Y80A Orai1. Each trace is the average of between 40 and 65 cells. (B) Images show movement of NFAT-GFP from cytosol to nucleus in an individual HEK293 cell transfected with STIM1 and Orai1 following continuous thapsigargin stimulation in 2 mM external Ca^2+^. Time in thapsigargin is indicated below each image. (C–E) Identical protocol to (B), but cells have been transfected with STIM1 and A73E Orai1 (C), STIM1 and W76A Orai1 (D), or STIM1 and Y80A Orai1 (E). (F) Graph comparing kinetics of NFAT-GFP movement into the nucleus (depicted as nuclear/cytosolic ratio) for the different conditions. Each trace is the average of between 11 and 15 individual cells. (G) Reporter gene expression in response to 160 nM LTC_4_, compared between RBL-1 cells transfected with Orai1 or A73E Orai1. STIM1 and GFP under an NFAT promoter were cotransfected in both cases. Left panels show transillumination images. (H) Aggregate data from five independent experiments. Data have been normalized to the percentage of cells expressing a reporter gene in Orai1-transfected cells. Error bars show SEM.

Bioinformatic analysis and site-directed mutagenesis studies have identified a calmodulin-binding domain on the N terminus of Orai1, between residues 68 and 90 [21]. Specific single point mutations within this domain alter calmodulin binding to Orai1, without affecting the activation of Orai1 channels [21]. We therefore examined the effects of these mutations on NFAT activation following CRAC channel opening. Transfection into HEK293 cells of either Orai1 or Orai1 constructs containing point mutations within the N terminus of Orai1 that suppressed calmodulin binding (A73E, W76A) [21] together with STIM1 resulted in robust store-operated Ca^2+^ entry following store depletion with the SERCA pump blocker thapsigargin (Figure 1A), and no differences in either Ca^2+^ release or Ca^2+^ entry rates were seen with the different constructs (Figure 1A). To measure NFAT activation, we cotransfected cells with an NFAT1(1-460)-GFP fusion protein [4], STIM1, and either wild-type Orai1 or one of the two mutant Orai1 constructs. Whereas robust NFAT migration into the nucleus occurred after stimulation with thapsigargin in cells transfected with wild-type Orai1 (Figures 1B and 1F), significantly less NFAT activation occurred in the presence of A73E Orai1 (Figures 1C and 1F) or W76A Orai1 (Figures 1D and 1F). We measured coupling between CRAC channels and gene expression through use of a reporter gene (GFP) driven by an NFAT promoter [4, 20, 22]. Stimulation of RBL-1 cells (transfected with Orai1, STIM1, and reporter gene) with the physiological trigger leukotriene C_4_, acting on cysteinyl leukotriene type I receptors, triggered GFP expression in ∼30% of the cells, and this was significantly reduced when A73E or W76A Orai1 was expressed instead (Figures 1G and 1H; data are normalized to cells transfected with nonmutated Orai1). These results suggest that mutations within the calmodulin-binding domain of Orai1 interfere with NFAT activation and subsequent gene expression. Mutation of a tyrosine residue to alanine (Y80A) in Orai1 revealed strong calmodulin association with the channel [21]. NFAT-GFP migration into the nucleus was reduced following stimulation with thapsigargin in cells cotransfected with Orai1Y80A and STIM1 (Figures 1E and 1F), as was gene expression (Figure 1H), despite the cytoplasmic Ca^2+^ signals being unaffected (Figure 1A). Further discussion of this mutant is presented below.

Calmodulin should be located close to the Orai1 channel pore if it is to detect the Ca^2+^ nanodomain near each open CRAC channel. Two arguments suggest that this is the case. First, Ca^2+^-dependent fast inactivation of CRAC channels, which is thought to be mediated by calmodulin [21, 23], is reduced by loading the cytoplasm with the fast Ca^2+^ chelator BAPTA, but not the slower EGTA, placing the Ca^2+^ binding site within ∼7 nm of the pore [24–26]. Consideration of the voltage dependence of fast inactivation combined with simulation of the local Ca^2+^ signal near the open channel puts the calmodulin site at <5.4 nm from the pore (see Figure S1 available online and associated text for details). Second, if calmodulin is indeed located close to the pore, then the rate and extent of fast inactivation should correlate with the speed of buildup and the size of the Ca^2+^ nanodomain, and this was indeed the case (Figure S1 and corresponding text).

**Figure 2:**
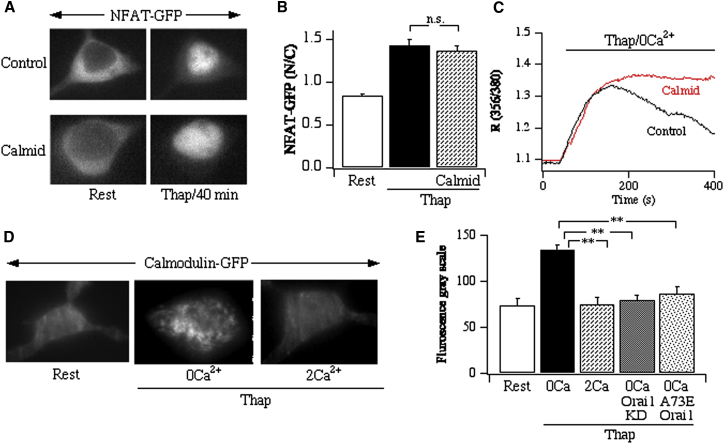
Calmodulin and Orai1 Interact in a Ca^2+^-Dependent Manner (A) NFAT activation occurs in the presence of calmidazolium (10 μM; 15 min pretreatment). (B) Aggregate data are summarized (rest, 21 cells; thapsigargin, 11 cells; calmidazolium + thapsigargin, 10 cells). (C) Calmidazolium (10 μM; 15 min pretreatment) slows Ca^2+^ extrusion by the Ca^2+^-calmodulin-dependent plasma membrane Ca^2+^ATPase pump. Cells were stimulated with thapsigargin in Ca^2+^-free solution. The decay of the Ca^2+^ signal is mainly due to Ca^2+^ATPase pump activity. (D) TIRF microscopy images following overexpression of nontagged Orai1, STIM1, and calmodulin-GFP. (E) Aggregate data plots of total GFP fluorescence in the evanescent field from several experiments as in (D). Each histogram is the mean of 10–13 cells (three preparations). Error bars show SEM. n.s., not significant; ^∗∗^p < 0.01.

When bound to the isoleucine-glutamine (IQ) domain of voltage-gated Ca^2+^ channels, calmodulin is shielded from pharmacological blockers [27]. If calmodulin is tethered to Orai1 or a closely related protein, we reasoned that it should also be relatively insensitive to such inhibitors. Consistent with this, NFAT migration into the nucleus was unaffected by the calmodulin inhibitor calmidazolium (Figures 2A and 2B). Following stimulation with thapsigargin in Ca^2+^-free solution, the decay of the Ca^2+^ signal is due mainly to the plasma membrane Ca^2+^ATPase pump [28], which is stimulated by calmodulin. The rate of Ca^2+^ clearance was slowed by calmidazolium (Figure 2C), confirming that calmidazolium was able to inhibit calmodulin in these cells. Does Ca^2+^ entry through Orai1 release a fraction of calmodulin from the channel? To test this, we carried out several independent experiments. First, we immunoprecipitated full-length Orai1-GFP and then blotted for endogenous calmodulin. Whereas strong interaction was found when the lysis buffer contained low Ca^2+^ (4 mM EGTA), the association between the proteins was significantly reduced, although not abolished, in high Ca^2+^ (Figures S2A and S2B). Conversely, after immunoprecipitation of calmodulin-GFP, we found significantly more association of endogenous Orai1 in low-Ca^2+^, but not high-Ca^2+^, lysis buffer (Figures S2C and S2D). We validated the use of the Orai1 antibody in the following manner. Knockdown of Orai1 using a small interfering RNA (siRNA) approach reduced CRAC channel activity by ∼70% [29] and reduced Orai1 expression by 62% ± 7% (Figure S2E). Hence, the anti-Orai1 antibody recognizes the protein.

We used tandem mass spectroscopy to confirm the identity of the gel band in low-Ca^2+^ buffer. Pull-down of calmodulin-GFP revealed the presence of Orai1 in lysis buffer containing 4 mM EGTA (Figure S2F), confirming the presence of both proteins in the gel band in low Ca^2+^. Following transfection with GFP alone, immunoprecipitation of GFP failed to reveal the presence of calmodulin in either 4 mM EGTA or 2 mM CaCl_2_ (Figure S2G), ruling out interaction between GFP and calmodulin. In a further set of experiments, we used total internal reflection fluorescence (TIRF) microscopy to measure the subplasmalemmal distribution of calmodulin before and then after store depletion. Coexpression of calmodulin-GFP and Orai1 (and STIM1) resulted in a general smearing of fluorescence within the evanescent field at rest (Figure 2D). Store depletion in the absence of external Ca^2+^ led to the formation of puncta of calmodulin-GFP, which resembled those formed by STIM1/Orai1 after store depletion [29]. Total GFP fluorescence in the evanescent field increased slightly after store depletion, suggesting modest recruitment of calmodulin-GFP along with redistribution within the field. Readmission of external Ca^2+^ led to a reduction in calmodulin-GFP puncta (Figure 2D; aggregate data are summarized in Figure 2E). Puncta were qualitatively less prominent after transfection with calmodulin-GFP alone (data not shown) or after knockdown of endogenous Orai1 (Figure 2E). Transfection of calmodulin-GFP, STIM1, and A73EOrai1 also resulted in fewer puncta (Figure 2E). Following expression of calmodulin-GFP, STIM1, and Orai1-cherry, we observed punctate-like structures of calmodulin-GFP and Orai1-cherry after store depletion in the absence of external Ca^2+^, which colocalized well, at least within the limit of resolution of confocal microscopy (Figure S3A). Addition of external Ca^2+^ disassembled the calmodulin-GFP puncta without affecting cherry-Orai1 clusters (Figure S3A). Studies with a myc-tagged Ca^2+^-insensitive mutant calmodulin protein (in which all four EF hands had been mutated [30]) provided further evidence in support of interaction between Orai1 and calmodulin (Figure S2H). This mutant protein is Ca^2+^ insensitive and therefore should remain associated with Orai1 even in high Ca^2+^. Following expression of Orai1-GFP and myc-tagged mutant calmodulin, immunoprecipitation of GFP revealed the presence of mutant calmodulin in both the absence and presence of Ca^2+^. Collectively, these results are consistent with the view that calmodulin is tethered close to Orai1 at low resting cytosolic Ca^2+^ levels and that a fraction is released from the channel following Ca^2+^ entry through CRAC channels.

Although our studies show Ca^2+^-independent association between Orai1 and calmodulin in low Ca^2+^, a previous study did not observe such an interaction [21]. We do not have an explanation for this, but subtle differences in experimental conditions might contribute. In addition, we cannot rule out an indirect association between calmodulin and Orai1, mediated through a bridging protein. Our observation of an apocalmodulin site on Orai1 or a closely associated protein would be consistent both with the kinetics of Ca^2+^-calmodulin-dependent fast inactivation of CRAC channels (which develops rapidly with an initial time constant of 10 ms) and with the observation that the mutant Ca^2+^-insensitive calmodulin protein reduces fast inactivation [23] (Figure S1).

**Figure 3:**
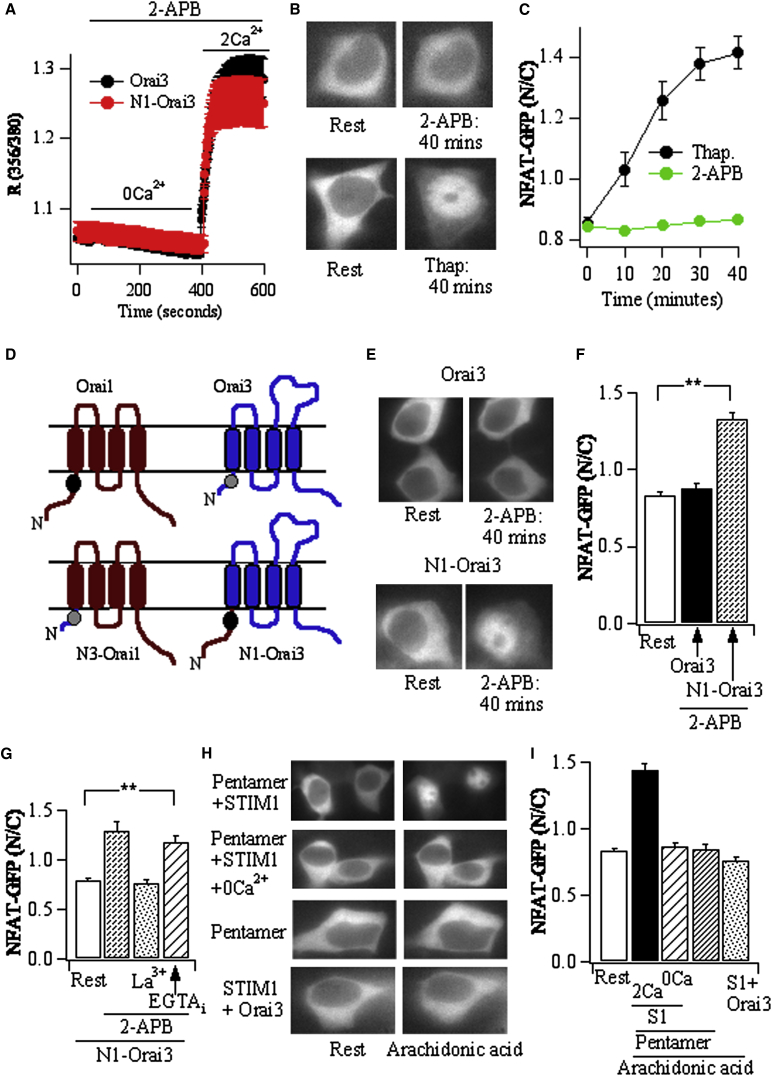
The N Terminus of Orai1 Couples Local Ca^2+^ to NFAT Activation (A) Following transfection with Orai3 and STIM1, acute exposure to 50 μM 2-APB evokes a large cytoplasmic Ca^2+^ rise (>50 cells). The N1-O3 construct also elicited a similar Ca^2+^ signal. (B) Despite eliciting robust Ca^2+^ entry, 2-APB fails to drive NFAT migration into the nucleus (upper panels). By contrast, stimulation with thapsigargin evokes clear NFAT movement in control cells from the same preparations. (C) Aggregate data plotting nuclear/cytosolic NFAT versus time, compared between cells activated with thapsigargin (11 cells, mock transfected and from the same preparations as those used for 2-APB) and cells activated with 2-APB (17 cells, transfected with STIM1 and Orai3). (D) Cartoon depicting chimeras that were synthesized. The dark, filled circle on Orai1 is calmodulin. It has been presented as less dark on Orai3, since the interaction appears weaker. (E) Whereas 2-APB fails to activate NFAT movement in cells expressing Orai3, it caused clear movement in a fraction of cells transfected with the N1-Orai3 construct. In these experiments, external Na^+^ was 10 mM (replaced with TRIS^+^). Cells were stimulated in low-Na^+^ (10 mM) solution containing 2 mM Ca^2+^. (F) Aggregate data from experiments in (E). Each bar represents >12 cells. (G) 2-APB-activated NFAT movement in N1-Orai3 cells is suppressed by the CRAC channel blocker La^3+^ (50 μM) but is unaffected by loading the cytoplasm with the slow Ca^2+^ chelator EGTA (five to nine cells for each condition). (H) NFAT migrates into the nucleus in response to stimulation with 8 μM arachidonic acid in cells expressing the pentamer and STIM1 in the presence of external Ca^2+^, but not when Ca^2+^ is absent or when STIM1 is not cotransfected. Arachidonic acid does not activate NFAT in cells transfected with Orai3 and STIM1. (I) Aggregate data from experiments as in (H). Each bar is the average of 20–35 cells. Error bars show SEM. ^∗∗^p < 0.01.

We asked whether the ability of Orai1 to activate NFAT was unique to the channel, or whether other store-operated Ca^2+^ channels could also recruit this pathway. Orai3 is a paralog of Orai1 and functions as a store-operated Ca^2+^ channel in expression systems [31–34]. Orai3 is gated directly by the pharmacological agent 2-aminoethoxydiphenyl borate (2-APB), which binds to the protein and dilates the channel pore [35–38]. Robust cytoplasmic Ca^2+^ signals were evoked when Ca^2+^ was applied to 2-APB-treated HEK293 cells coexpressing Orai3 and STIM1 (Figure 3A), and these were substantially smaller in nontransfected cells (data not shown). The Ca^2+^ signal to 2-APB in Orai3-expressing cells was similar to that evoked by store depletion in Orai1-expressing cells (Figure 1A), yet the former consistently failed to activate NFAT (Figures 3B and 3C). This was not due to an inhibitory effect of 2-APB on the downstream signaling pathway, because raising bulk cytoplasmic Ca^2+^ with a high concentration of ionomycin caused robust NFAT activation after exposure to 2-APB (Figure S3B). When gated by 2-APB, Orai3 channels are permeable to both Na^+^ and Ca^2+^ [37, 38]. Na^+^ flux through the channels would depolarize the membrane potential, thus reducing the size and radial spread of the local Ca^2+^ signal. To circumvent this, we applied 2-APB in low-Na^+^ (10 mM) external solution. However, NFAT still did not migrate to the nucleus (Figure 3E, upper panel). Although sequence alignment revealed several conserved residues within the N terminus calmodulin-binding domain of Orai1 with a corresponding stretch in Orai3, the N terminus of Orai3 is considerably shorter than Orai1. We reasoned that a calmodulin-binding domain in Orai3 [39], if functional, would be less efficacious than Orai1 in coupling local Ca^2+^ signals to NFAT. We therefore made two chimeras: in one, we replaced the N terminus of Orai1 with that from Orai3 (N3-Orai1; Figure 3D), and in the other, we replaced the N terminus of Orai3 with that from Orai1 (N1-Orai3; Figure 3D). The N3-Orai1 construct failed to produce a clear Ca^2+^ signal following stimulation with thapsigargin, consistent with a previous report of poor functional expression [40]. By contrast, N1-Orai3 channels responded to 2-APB by generating detectable Ca^2+^ signals (Figure 3A). Whereas 2-APB failed to stimulate NFAT movement in those cells transfected with Orai3 channels (Figures 3E and 3F), NFAT activation occurred when N1-Orai3 channels were expressed instead (Figures 3E and 3F), although fewer transfected cells responded (∼2-fold less) compared with stimulation with thapsigargin in cells overexpressing STIM1 and Orai1. NFAT activation to 2-APB in N1-Orai3-expressing cells was suppressed by omission of external Ca^2+^ or by block of the Orai3 channels with La^3+^ (Figure 3G) but was unaffected by loading the cytoplasm with the slow Ca^2+^ chelator EGTA (Figure 3G). The presence of the N terminus of Orai1 therefore helps enable Orai3 to activate cytoplasmic NFAT via local Ca^2+^ entry.

Fast inactivation of CRAC channels is thought to require Ca^2+^-dependent binding of calmodulin to the N terminus of Orai1 [21]. Tethered calmodulin also appears necessary for activation of NFAT, and this would require Ca^2+^-dependent dissociation from Orai1, presumably from the apocalmodulin site. CaV1.3 channels have two distinct calmodulin-binding sites: a vestigial EF hand region upstream of the IQ domain on the C terminus, and an N-terminal spatial Ca^2+^ transforming element module. Ca^2+^-calmodulin is able to shuttle between the two, with different effects on channel activity [41]. It is conceivable that there are two calmodulin-binding sites on the N terminus of Orai1 and that mutations in the Ca^2+^-calmodulin binding site (e.g., A73) destabilize the apocalmodulin region. Alternatively, specific mutations with the calmodulin-binding domain might result in weak association between Orai1 and a bridging protein that brings calmodulin close to the Orai1 pore. In the Y80A Orai1 mutant, calmodulin remains bound in both low and high Ca^2+^ (Figure S2I), and this is associated with strong, fast inactivation of the channel. The weaker activation of NFAT with this construct could therefore reflect a slower dissociation rate of calmodulin from Orai1 and/or the reduction in local Ca^2+^ influx following opening of the mutant channel [21].

Can the presence of Orai1 within a heteromultimeric channel complex (containing Orai1 and non-Orai1 components) confer the ability to couple local Ca^2+^ to NFAT activation, when the other subunits within the multimer are ineffective? To test this, we transfected cells with an arachidonic acid-gated non-store-operated Ca^2+^ channel, a pentamer of three Orai1 and two Orai3 subunits, along with the regulator STIM1 [33]. Stimulation with arachidonic acid resulted in robust NFAT migration into the nucleus (Figures 3H and 3I). No detectable movement occurred in the absence of external Ca^2+^ or when the pentamer was expressed without STIM1 (Figures 3H and 3I). Arachidonic acid failed to stimulate NFAT movement in cells cotransfected with STIM1 and Orai3 (Figures 3H and 3I) or with STIM1 and Orai1 (zero of six cells showed movement). Inclusion of Orai1 in a heteromeric channel complex thus imparts the ability to transduce local Ca^2+^ signals into NFAT activation. We asked whether the preferential coupling of Orai1 to NFAT was due exclusively to tethered calmodulin. Pull-down experiments revealed association of calmodulin with Orai3 (Figure S3C), but this was significantly less than that seen with Orai1. Although the N1-Orai3 protein had more associated calmodulin than Orai3, this was clearly less than for Orai1 (Figure S3C). We therefore reasoned that an additional mechanism contributed to effective coupling between Orai1 and NFAT.

**Figure 4:**
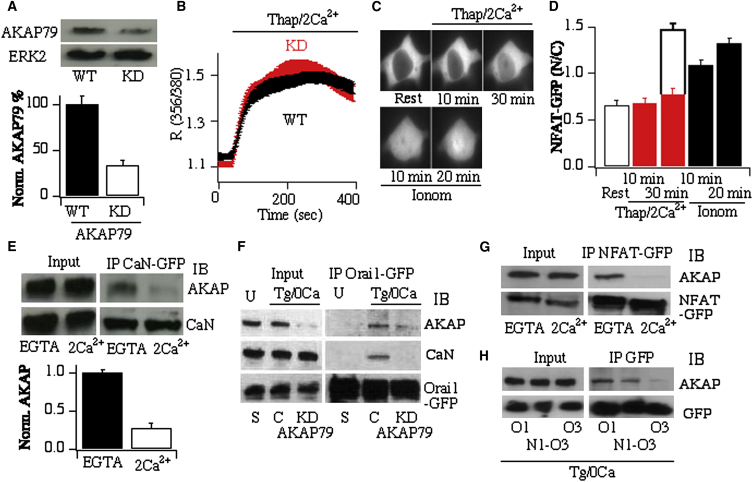
Formation of a Store-Dependent Signaling Complex (A) Western blot showing that siRNA against AKAP79 reduces protein expression. KD, knockdown. (B) Knockdown (KD) of AKAP79 fails to alter the Ca^2+^ signal to thapsigargin (2 μM) in external Ca^2+^ (average of >50 cells per condition). (C) NFAT-GFP nuclear migration is reduced after knockdown of AKAP79. Subsequent stimulation with ionomycin (5 μM) in the same cell led to strong movement to the nucleus. (D) Aggregate data from several experiments. Red bars denote cells treated with siRNA against AKAP79. The open bar at 30 min shows the response to thapsigargin in mock-transfected cells at the same time point. Black bars denote the response to ionomycin, applied 10 or 20 min after thapsigargin treatment for 30 min in cells treated with siRNA against AKAP79. (E) Coimmunoprecipitation experiments show strong interaction between AKAP79 and calcineurin in low-Ca^2+^, but not high-Ca^2+^, lysis buffer. Calcineurin-GFP was pulled down, and immunoblot (IB) was for endogenous AKAP79. (F) Immunoprecipitation of Orai1-GFP was associated with little calcineurin in unstimulated cells (labeled U), but the extent of interaction increased after store depletion in Ca^2+^-free external solution. Interaction was reduced after knockdown of AKAP79. Cells were exposed to thapsigargin in Ca^2+^-free solution for 5 min before lysis in 4 mM EGTA-containing buffer. S denotes transfection with scrambled siRNA; C represents control cells (stimulated with thapsigargin in Ca^2+^-free solution) for the AKAP knockdown data set. (G) Blots show association of NFAT-GFP with AKAP79 in low-Ca^2+^ (EGTA), but not high-Ca^2+^, lysis buffer. In (G) and (H), gels are representative of three independent experiments. (H) Pull-down experiments comparing extent of interaction between Orai1, N1-Orai3, and Orai3 channels (all GFP-tagged) and AKAP79. Error bars show SEM.

Drawing on findings from voltage-gated Ca^2+^ channels, where calcineurin can be held close to CaV1.2 [42], we considered that calcineurin might bind to Orai1. Coimmunoprecipitation studies failed to reveal the presence of calcineurin when Orai1-GFP was pulled down in resting, untreated cells (Figure 4F, labeled U), arguing against association of calcineurin to Orai1 under these conditions. An elegant study in hippocampal neurons demonstrated that the scaffold protein AKAP79 recruited calcineurin to L-type channels [5]. We therefore explored the possibility that AKAP79 orchestrated the reversible formation of an Orai1-calcineurin complex following store depletion. Knockdown of AKAP79 using a siRNA approach resulted in ∼65% reduction of protein expression (Figure 4A). The Ca^2+^ signal evoked by thapsigargin was unaffected by the decreased levels of AKAP79 (Figure 4B), but NFAT activation was significantly impaired (Figures 4C and 4D). Even 30 min after stimulation, little NFAT had migrated into the nucleus (Figure 4D, red bars; the open bar in Figure 4D at 30 min denotes NFAT movement in mock-transfected cells under similar conditions). High, nonphysiological levels of cytoplasmic Ca^2+^ activate NFAT independent of local Ca^2+^ entry in HEK cells [4]. NFAT migration was recovered in AKAP79-deficient cells following a large elevation of bulk Ca^2+^ independent of Orai1 by stimulating cells with a high concentration of the Ca^2+^ ionophore ionomycin (Figure 4D). Therefore, loss of AKAP79 does not impair the ability of Ca^2+^ to recruit the NFAT pathway per se; it inhibits Orai1 coupling to NFAT. Pull-down of calcineurin-GFP revealed the presence of AKAP79 in low Ca^2+^ but significantly less association in high Ca^2+^ (Figure 4E). This could reflect Ca^2+^-dependent reduction in calcineurin affinity for AKAP79 or a redistribution of the bound phosphatase to other substrates when Ca^2+^ is raised. Whereas pull-down of Orai1-GFP revealed little association of calcineurin in nonstimulated cells, interaction was revealed after store depletion with thapsigargin (Figure 4F). Knockdown of AKAP79 resulted in loss of interaction between Orai1 and calcineurin after store depletion (Figure 4F). AKAP79 therefore brings calcineurin close to Orai1, thereby juxtaposing the phosphatase with its activator calmodulin. We also found some association of NFAT-GFP with AKAP79 in resting cells, and this was reduced in elevated Ca^2+^ (Figure 4G). NFAT dephosphorylation occurred under these conditions, which might have resulted in dissociation or reorganization of a resting NFAT complex [43] and the loss of interaction with AKAP79. By contrast with Orai1, Orai3 failed to interact with AKAP79 (Figure 4H) or calcineurin (Figure S3D) to any detectable extent. The N1-Orai3 protein rescued, albeit partially, interaction between the channel and AKAP79 (Figure 4H).

Whereas overexpression of wild-type AKAP79 had no effect on either thapsigargin-evoked Ca^2+^ signals or on the ability of NFAT-GFP to migrate to the nucleus (Figures S4A–S4C), expression of an AKAP79 mutant (ΔPIX-AKAP79) that cannot bind calcineurin [42] inhibited NFAT accumulation in the nucleus without altering the Ca^2+^ signal (Figures S4A–S4C). Both proteins were expressed at similar levels (Figure S4D). Pull-down experiments confirmed that ΔPIX-AKAP79 failed to interact with calcineurin (Figure S4E). Furthermore, following knockdown of AKAP79, transfection with wild-type AKAP79, but not ΔPIX-AKAP79, rescued NFAT activation (Figures S4B and S4C).

A major question in Ca^2+^ signaling is how Ca^2+^ from different sources activates distinct cellular responses. In many cell types in various species, local Ca^2+^ signals near open Ca^2+^ channels selectively stimulate downstream targets, an effect often mediated through calmodulin. Although Ca^2+^ dissociates relatively slowly from the C-lobe of calmodulin, we calculate a diffusion distance of ∼0.5–0.8 μm before Ca^2+^ is released from the protein. This prompts the question: how does a downstream target located a few micrometers away from the plasma membrane distinguish Ca^2+^-calmodulin activated by local Ca^2+^ near one class of Ca^2+^ channel from that generated by other channels or from the relatively large amounts of free calmodulin in the cytoplasm? And how does Ca^2+^-calmodulin from one Ca^2+^ source activate one target and not others? Our findings shed new insight into this universal decoding problem by revealing the presence of a store-dependent mechanism that brings the activator (calmodulin) into close proximity to the intermediary (calcineurin) and executor (NFAT). Calmodulin is tethered to Orai1 or a closely associated protein and is therefore held close to the source of Ca^2+^. This pool of calmodulin has privileged access to calcineurin because store depletion recruits AKAP79 with attached calcineurin to Orai1. This form of coincidence detection, requiring two conditions to be satisfied (tethered calmodulin and store-dependent association of AKAP79, the latter bringing calcineurin and NFAT to Orai1), provides a mechanism for ensuring that only local Ca^2+^ entry via store depletion will activate the pathway. Ca^2+^ release from stores or local Ca^2+^ entry through other channels such as Orai3 will not be effective because AKAP79 is not recruited to, and there is less calmodulin associated with, Orai3. This dual requirement for tethered calmodulin and AKAP in mast cells prevents physiological fluctuations in cytosolic Ca^2+^ from activating NFAT in the absence of store depletion. Large, nonphysiological bulk rises in Ca^2+^ can activate NFAT, even in the absence of AKAP79 (Figure 4D), by raising cytoplasmic Ca^2+^ sufficiently to match the local physiological Ca^2+^ rise near Orai1. Given that impaired calcineurin-NFAT signaling is linked to various disorders in nonexcitable cells, including autoimmune disease, osteoporosis, Down syndrome, and possibly cancer [1], the privileged pathway between Orai1 and NFAT identifies the channel as a potential new target for management of these diseases.

## Supplemental Information

Document S1. Figures S1–S4 and Supplemental Experimental Procedures

Document S2. Article plus Supplemental Information
