# Supplementary material for: Dynamic Assembly of a Membrane Signaling Complex Enables Selective Activation of NFAT by Orai1
Source: Curr Biol. 2014 Jun 16;24(12):1361–8. doi: 10.1016/j.cub.2014.04.046 (PMC4062936; doi:10.1016/j.cub.2014.04.046)
Supplement: Document S1. Figures S1–S4 and Supplemental Experimental Procedures [file mmc1.pdf]

Current Biology, Volume *24*
Supplemental Information

# Dynamic Assembly of a Membrane Signaling Complex Enables Selective Activation of NFAT by Orai1

Pulak Kar, Krishna Samanta, Holger Kramer, Otto Morris, Daniel Bakowski, and Anant B. Parekh

Supplemental Figure 1

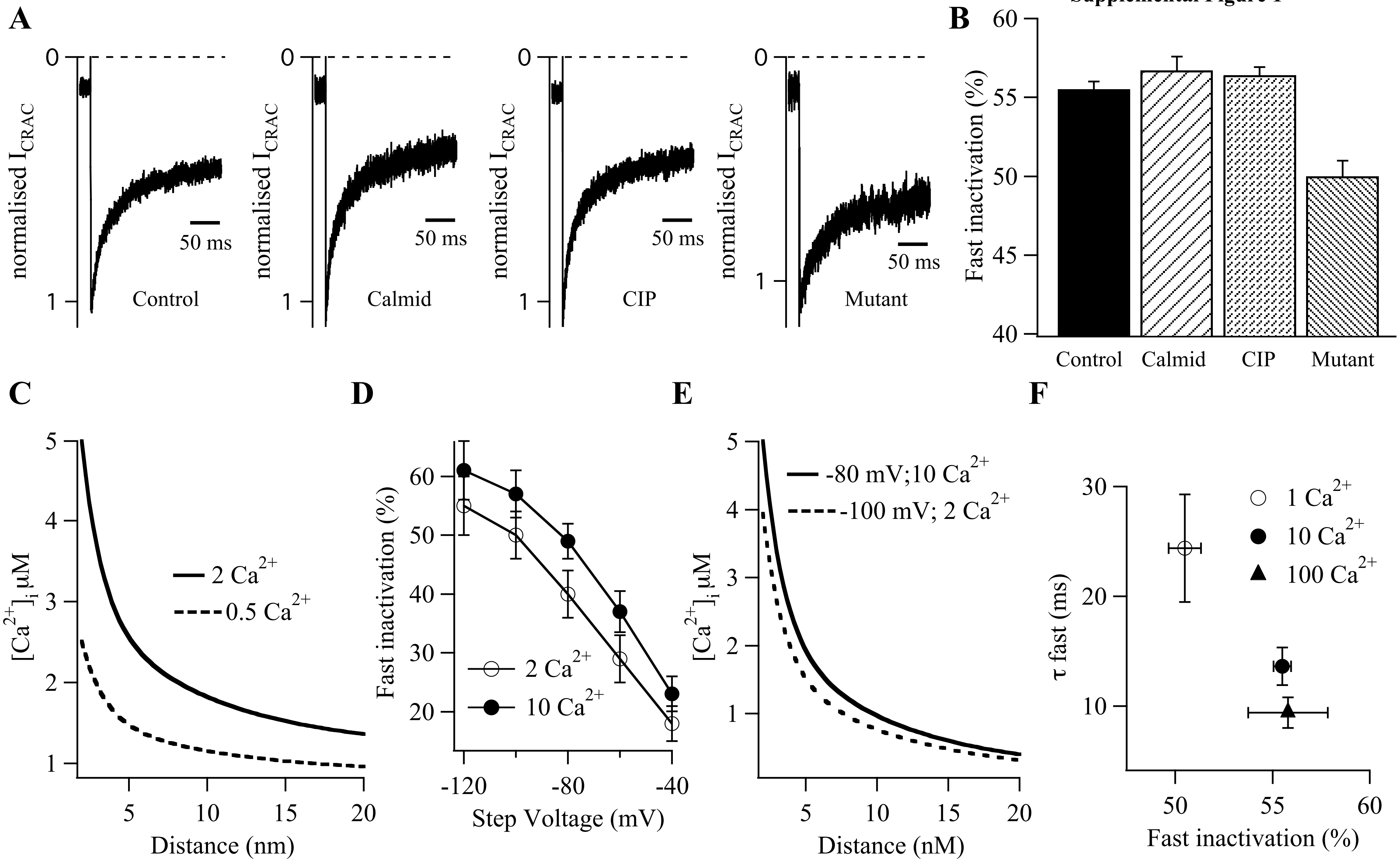

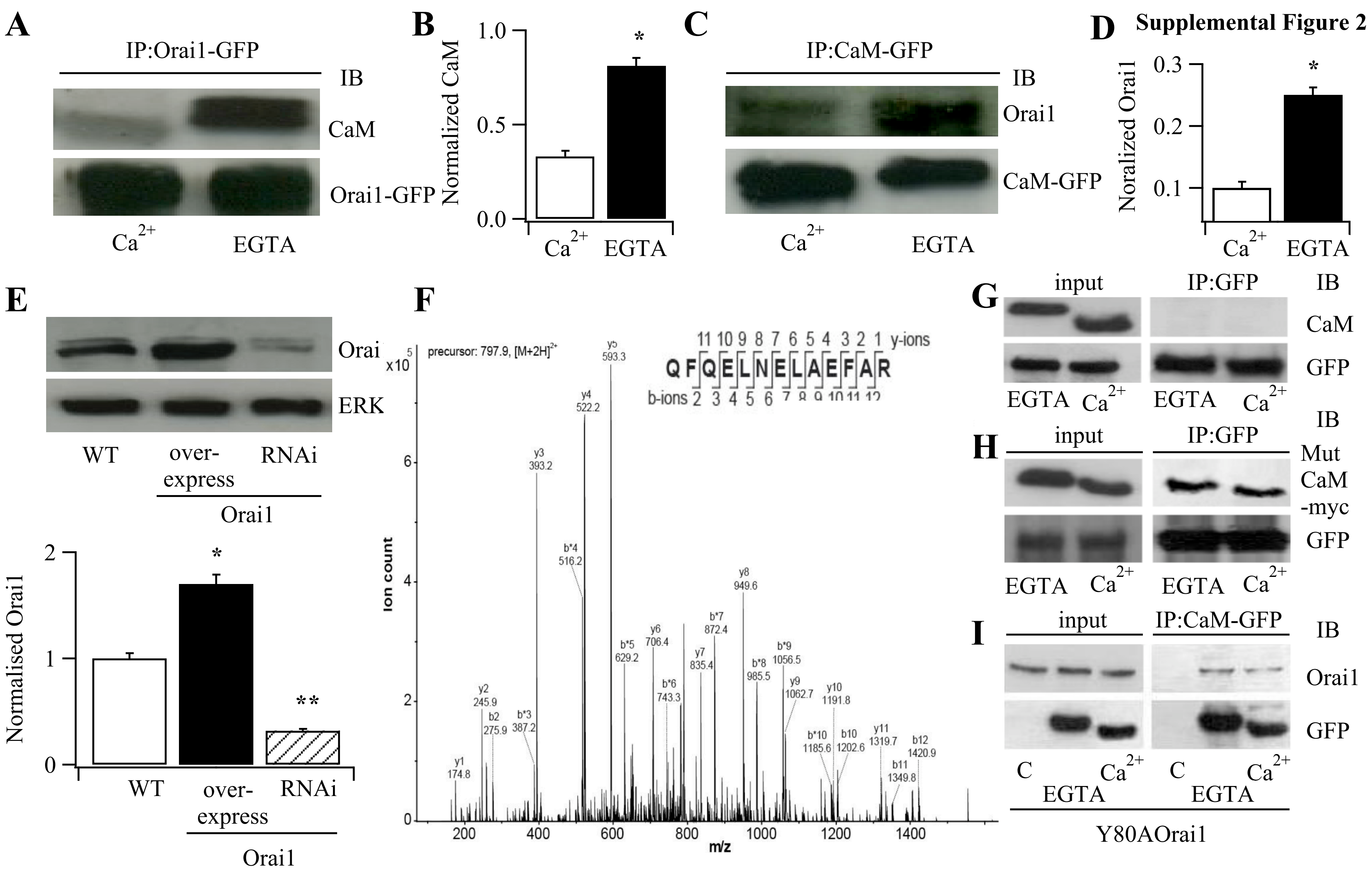
Supplemental Figure 2
A
IP:Orai1-GFP
IB
CaM
Orai1-GFP
Ca2+
EGTA
B
Normalized CaM
1.0
0.5
0.0
Ca2+
EGTA
C
IP:CaM-GFP
IB
Orai1
CaM-GFP
Ca2+
EGTA
D
Noralized Orai1
0.3
0.2
0.1
Ca2+
EGTA
E
Orai
ERK
WT
over-express
RNAi
Orai1
Normalised Orai1
2
1
0
WT
over-express
RNAi
Orai1
F
precursor: 797.9, [M+2H]2+
11 10 9 8 7 6 5 4 3 2 1 y-ions
QFQELNELAEFAR
b-ions 2 3 4 5 6 7 8 9 10 11 12
Ion count
x10^5
m/z
G
input
IP:GFP
IB
CaM
GFP
EGTA
Ca2+
EGTA
Ca2+
H
IB
input
IP:GFP
Mut CaM -myc
GFP
EGTA
Ca2+
EGTA
Ca2+
I
input
IP:CaM-GFP
IB
Orai1
GFP
C
EGTA
Ca2+
C
EGTA
Ca2+
Y80AOrai1

**Supplemental Figure 3**

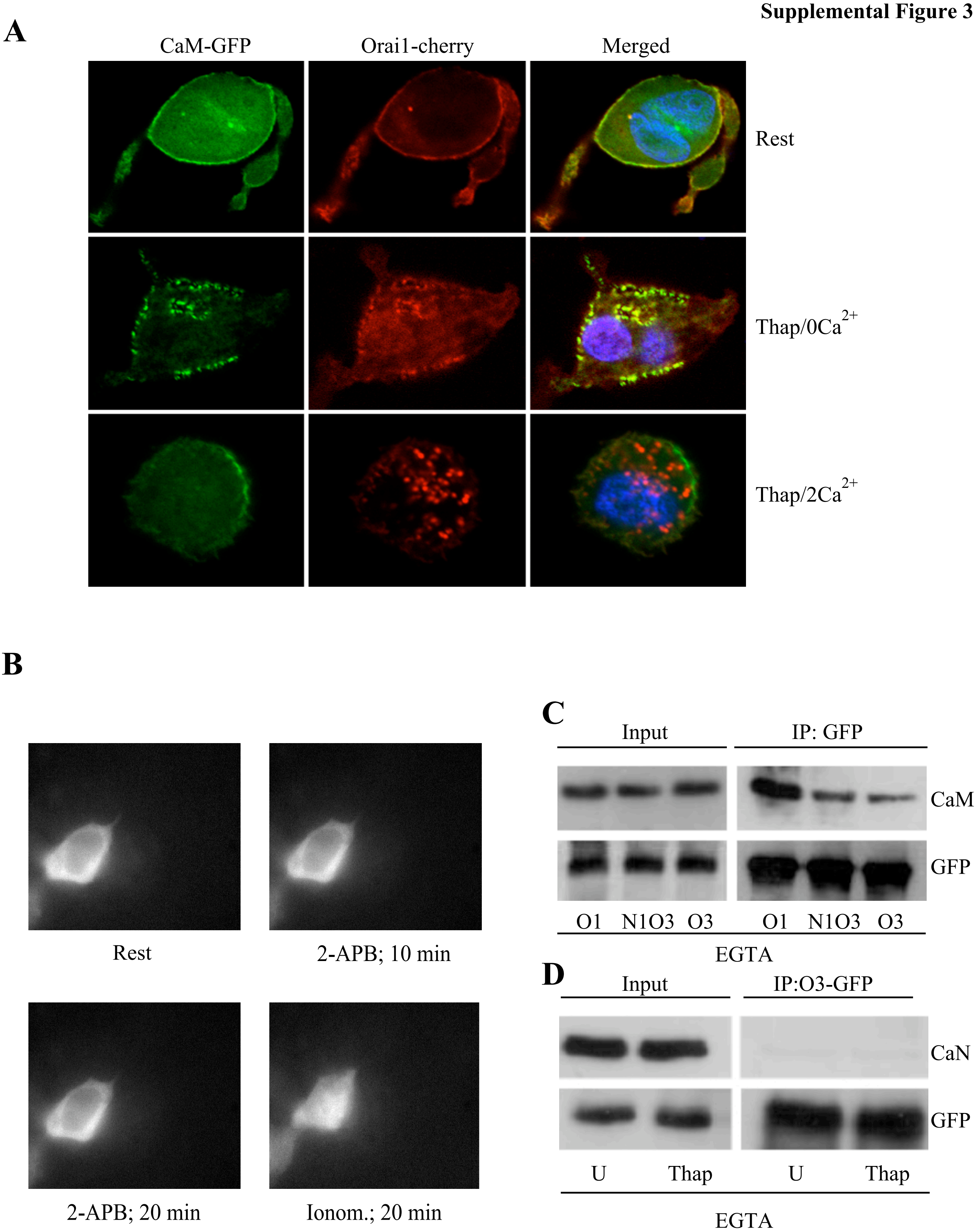

**Supplemental Figure 4**

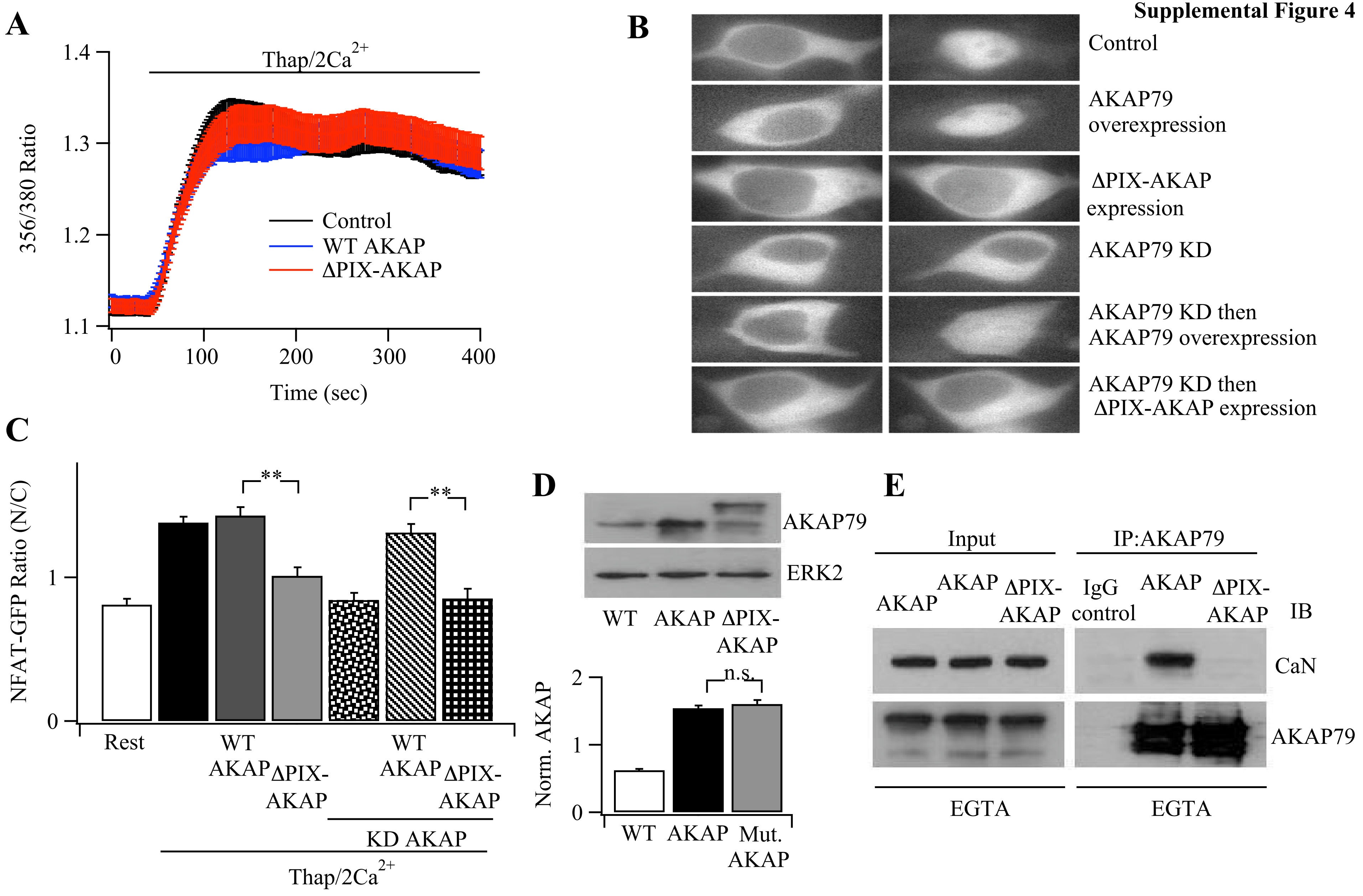

**SUPPLEMENTAL**

**SUPPLEMENTAL FIGURE LEGEND**

Supplemental Figure 1, related to Figure 2. Tethered calmodulin is close to the CRAC channel pore. A, Cells were dialysed with pipette solution containing $InsP_3$ and 10 mM EGTA and voltage steps applied for 250 ms to -100 mV from a holding potential of 0 mV. Calmid denotes calmidazolium (20 μM), CIP is calmodulin inhibitory peptide (20 μM) and mutant is a double mutant calmodulin in which $Ca^{2+}$ binding sites in both N- and C-lobes have been lost.

Cells were pre-treated with calmidazolium for 15 minutes and the inhibitor was also added to the pipette solution. CIP was dialyzed into the cell via the pipette. For mutant, recordings were made ~ 40 hours after transfection. Cells were co-transfected with eGFP to identify transfected cells. B, Aggregate data from several experiments are compared. Each bar is the average of 8-14 cells. C, Simulation of the spatial profile of local $Ca^{2+}$ as a function of distance from the CRAC channel pore under conditions where cytosolic $Ca^{2+}$ is free to rise. $[Ca^{2+}]_{local} = (i_{Ca}/4*\pi*F*D_{Ca}*r) + [Ca^{2+}]_{steady\ state}$. The latter was measured (using fura 2) as ~ 1.0 μM following stimulation with thapsigargin in 2 mM $Ca^{2+}$ and ~ 0.8 μM in 0.5 mM $Ca^{2+}$. Values for $i_{Ca}$ were -2.91 fA (2 mM $Ca^{2+}$, estimated from noise analysis) and -1.247 fA (0.5 mM $Ca^{2+}$, obtained by scaling the macroscopic current to that seen in 2 mM $Ca^{2+}$). $D_{Ca}$ is taken as 300 $\mu m^2/s$. r is the distance from the pore. D, Voltage-dependence of fast inactivation is compared between 2 mM and 10 mM external $Ca^{2+}$. CRAC channels were activated by dialysis with $InsP_3$ and 10 mM EGTA. Hyperpolarizing pulses were applied to different voltages from a holding potential of 0 mV at 0.5 Hz. E, Simulation of local $Ca^{2+}$ following flux through CRAC channels at -80 mV in 10 mM $Ca^{2+}$ or -100 mV in 2 mM $Ca^{2+}$, based on the recording conditions in panel D (high intracellular EGTA). In high

intracellular $Ca^{2+}$ buffer, $[Ca^{2+}]_{local} = (i_{Ca}/4*\pi*F*D_{Ca}*r)*exp(-r/\lambda)$, where $\lambda$ is the mean path length of $Ca^{2+}$ (calculated as 120 nm in 10 mM EGTA). F, Graph plots the kinetics of fast inactivation against extent of fast inactivation following CRAC channel opening under the different external $Ca^{2+}$ concentrations shown. Each point is the average of between 7 and 13 cells.

Supplemental Figure 2, related to Figure 2. $Ca^{2+}$-dependent interaction between calmodulin (CaM) and Orai1. A, Following immunprecipitation of Orai-GFP on GFP-coated beads, samples were blotted for calmodulin. Lysis buffer contained either 2 mM $CaCl_2$ or 4 mM EGTA. B, Aggregate data from 4 independent experiments are compared. CaM immunoblots were normalised to Orai1-GFP levels. C, Following immunoprecipitation of calmodulin-GFP, samples were blotted for Orai1 for the two conditions shown. D, Aggregate data are summarised from 3 separate experiments. Orai1 immunoblots were normalised to CaM-GFP levels. E, The Orai1 antibody detects changes in the levels of Orai1. The histogram summarises aggregate data from 3 independent experiments. F, Identification of Orai1 by tandem mass spectrometry (LC-MS/MS). Fragmentation spectrum of a tryptic peptide leading to identification of human Orai1 (269-281) by the MASCOT algorithm with an ions score of 101; annotations: *, loss of ammonia/ deamination (-17 Da), °, loss of water/ dehydration (-18 Da). G, Following transfection with GFP alone, pulldown of GFP failed to reveal the presence of calmodulin in either EGTA or $Ca^{2+}$-containing lysis buffer. H, $Ca^{2+}$-insensitive calmodulin is associated with Orai1. Cells were transfected with Ora1-GFP and myc-tagged mutant calmodulin (an asparate residue in each of the four EF hands had been mutated to an alanine) plasmids. After pulldown of GFP, samples were blotted for myc-tagged calmodulin in lysis buffer containing either 4 mM EGTA or 2 mM $Ca^{2+}$. I, Y80A Orai1 binds calmodulin in the absence

(EGTA) and presence (2 mM) of $Ca^{2+}$ in lysis buffer. C denotes control; cells that had not been transfected with CaM-GFP. These cells were then treated in the same way as those that had been transfected, and this included pulldown on GFP-coated beads. As the 'C' group had no CaM-GFP, this is a negative control.

Supplemental Figure 3, related to Figure 2 and Figure 3. 2-APB does not impair NFAT activation following a rise in $Ca^{2+}$ evoked by a high concentration of ionomycin (5 μM). A, Confocal microscope images show distribution of calmodulin-GFP and Orai1-cherry in non-stimulated RBL cells, after store depletion with thapsigargin in $Ca^{2+}$-free solution and 4 minutes after readmission of external $Ca^{2+}$ to cells treated with thapsigargin in $Ca^{2+}$-free solution. B, 2-APB failed to elicit NFAT-GFP movement to the nucleus in cells overexpressing Orai3. However, stimulation with ionomycin (in the presence of 2-APB) resulted in strong nuclear migration. C, Following transfection, GFP-tagged Orai1, N1-Orai3 or Orai3 were immunoprecipitated and blotted for CaM. Lysis buffer contained 4 mM EGTA. D, Following immuno-precipitation of Orai3-tagged GFP, samples were blotted for calcineurin in either untreated (non-stimulated; labelled U) cells or after store depletion following exposure to thapsigargin (2 μM) in $Ca^{2+}$-free solution for 5 minutes (labelled Th/0). Lysis buffer contained 4 mM EGTA.

Supplemental Figure 4, related to Figure 4. Expression of ΔPIX-AKAP79 impairs CRAC channel-dependent activation of NFAT. A, Cytoplasmic $Ca^{2+}$ signals to thapsigargin are unaffected by overexpression of wild type AKAP79 or expression of ΔPIX-AKAP79-cherry. B, Nuclear accumulation of NFAT-GFP is compared for the different conditions. AKAPKD refers to knockdown of AKAP79. 24 hours after knockdown, cells were transfected with either wildtype AKAP79 or ΔPIX-AKAP79-cherry. C, Aggregate data from several

experiments are compared. Each bar is the mean of between 6 and 11 cells. D, Western blot compares expression of AKAP79 in wildtype cells (non-transfected), after overexpression of AKAP79 and after expression of ΔPIX-AKAP79-cherry. Note the two bands in the latter case, reflecting endogenous AKAP and tagged recombinant protein. E, Pulldown experiments show that AKAP79-YFP is associated with calcineurin in the absence of external $Ca^{2+}$ but no interaction is seen when ΔPIX-AKAP79-CFP is expressed instead. Lysis buffer contained 4 mM EGTA. IgG control is shown, which represents pull down of AKAP79-YFP onto non-GFP beads.

## SUPPLEMENTAL
## EXPERIMENTAL PROCEDURES

**Cell Culture and Transfection**-HEK293 and rat basophilic leukemia (RBL-1) cells were bought from ATCC and were cultured (37 °C, 5% $CO_2$) in Dulbecco's modified Eagle medium with 10% fetal bovine serum, 2 mM L-glutamine and penicillin-streptomycin, as previously described[S1]. HEK293 cells were transfected using the lipofectamine method. RBL-1 cells were transfected using the AMAXA system. RBL-1 cells were used for reporter gene expression studies (Fig. 1G, H), measurement of fast inactivation of endogenous CRAC channels (Supplementary Figure 1) and confocal microscopy of calmodulin-GFP and Orai-cherry (Supplementary Figure 3A). All other data were obtained with HEK293 cells.

**cDNA Constructs-**Orai1 and STIM1 were purchased from Origene. STIM1-YFP was a gift from Dr Tobias Meyer (Stanford). Orai1-GFP, Orai3-GFP and calmodulin mutant were provided by Dr. James Putney (NIEHS). NFAT-GFP was provided by Dr Paul Worley (Johns Hopkins). GFP driven by an NFAT promoter was from Dr Yuriy Usachev (Iowa). Orai1-cherry was from Dr Alexey Tepikin (Liverpool). AKAP79-YFP was kindly provided by Dr Mark Dell'Acqua (Colorado). The AKAP mutant ΔPIX variant (deletion of PIAIIIT),

which cannot bind calcineurin, was from Dr Dell'Acqua and was tagged with either CFP or cherry. The Orai1-Orai3 pentamer was kindly provided by Dr Trevor Shuttleworth (Rochester, NY). Orai1 mutants were generated by site-directed mutagenesis and verified by sequencing. A73E was initially generated using a GeneArt kit from Invitrogen and then confirmed by purchase of an A73E mutant from Mutagenex. W76A and Y80A, and the mutant myc-tagged calmodulin construct were obtained from Mutagenex.

**$Ca^{2+}$ imaging**-$Ca^{2+}$ imaging experiments were carried out at room temperature using the IMAGO CCD camera-based system from TILL Photonics, as described previously[S1]. Cells were alternately excited at 356 and 380 nm (20 msec exposures) and images were acquired every 2 seconds. Images were analysed offline using IGOR Pro for Windows. Cells were loaded with Fura 2-AM (2 μM) for 40 minutes at room temperature in the dark and then washed three times in standard external solution of composition (in mM) NaCl 145, KCl 2.8, $CaCl_2$ 2, $MgCl_2$ 2, D-glucose 10, HEPES 10, pH 7.4 with NaOH. Cells were left for 15 minutes to allow further deesterification. $Ca^{2+}$-free solution had the following composition (in mM) NaCl 145, KCl 2.8, $MgCl_2$ 2, D-glucose 10, HEPES 10, EGTA 0.1, pH 7.4 with NaOH). Low $Na^+$ external solution contained (in mM) NaCl 10, TRIS base 135, KCl 2.8, $CaCl_2$ 2, $MgCl_2$ 2, D-glucose 10, HEPES 10, EGTA 0.1, pH 7.4 with HCl).

***$I_{CRAC}$* recordings-**Patch-clamp experiments were conducted in the tight-seal whole-cell configuration at room temperature (20-24$^0$C) as previously described[S2]. Sylgard-coated, fire-polished pipettes had d.c. resistances of 4.2-5.5MΩ when filled with standard internal solution that contained (in mM): $Cs^+$ glutamate 145, NaCl 8, $MgCl_2$ 1, Mg-ATP 2, Ethylene glycol-bis(b-aminoethyl ether)-N,N,N',N',-tetraacetic acid (EGTA) 10, $InsP_3$ (0.03), HEPES 10, pH 7.2 with CsOH. A correction of 10 mV was applied for the subsequent liquid

junction potential that arose from this glutamate-based internal solution. The composition of the extracellular solution was (in mM): NaCl 145, KCl 2.8, $MgCl_2$ 2, CsCl 10, D-glucose 10, HEPES 10, pH 7.4 with NaOH. In some experiments, 1, 10 mM or 100 mM $CaCl_2$ was added (described in manuscript). 100 mM $CaCl_2$ solution contained 10 mM NaCl.

$I_{CRAC}$ was measured by applying voltage ramps (-100 to +100 mV in 50 msec) at 0.5 Hz from a holding potential of 0 mV. For fast inactivation, step pulses (250 msec duration) were applied from 0 mV to -100 mV every 2 seconds. Currents were filtered using an 8-pole Bessel filter at 2.5 kHz and digitised at 100 ms. Inactivation was determined by dividing the steady state current during the hyperpolarising pulse (measured after 240 ms) by the initial current (measured after 1 ms). Capacitative currents were compensated before each ramp by using the automatic compensation of the EPC 9 -2 amplifier. Leak currents were subtracted by averaging 2-3 ramp currents obtained just before $I_{CRAC}$ had started to develop, and then subtracting this from all subsequent currents.

**TIRF microscopy**-Cells were transfected with calmodulin-GFP and Orai1 24-36 hours prior to recording. TIRF recordings were carried out as described[S3].

**EGTA-AM loading-**Cells were loaded with EGTA by incubation for 45 minutes with EGTA-AM as described [S4].

**Nuclear NFAT1-GFP**-NFAT1-GFP levels in the cytosol and nucleus was measured using the IMAGO charge-coupled device camera-based system from TILL Photonics, with a x100 oil immersion objective (numerical aperture 1.3). Regions of interest of identical size were drawn in the cytosol and nucleus of each cell and fluorescence computed. Nuclear localization was confirmed by co-staining with a nuclear dye (DAPI), as described[S4]. Unless otherwise indicated we calculated the nuclear/cytosolic ratio of NFAT-GFP.

**Gene reporter assay**-24-36 hours following transfection with the EGFP-based reporter plasmid driven by an NFAT promoter, cells were stimulated with leukotriene $C_4$ (160 nM, 40 minutes) and the % of cells expressing EGFP measured[S4]. Gene expression was defined as fluorescence 3xSD> cell autofluorescence, measured in non-transfected cells. Cells were stimulated in culture medium and maintained in the incubator.

**Co-immunoprecipitation and Western blotting-**Twenty four hours after transfection, HEK293 cells were treated with 2 μM thapsigargin in $Ca^{2+}$ free external solution for 7 minute and then lysed in 50 mM Tris-HCl (pH 7.5), 150 mM NaCl, 1% Triton X-100, and protease inhibitors with either 2 mM $CaCl_2$ (for $Ca^{2+}$ condition) or 4 mM EGTA (for $Ca^{2+}$ free condition) for 15 min, as described by Mullins et al. [S5]. Lysates were spun at 12000 × g for 10 min, and the supernatant was used for immunoprecipitation reaction (anti-GFP agarose beads) at 4°C. After washing four times with ice cold lysis buffer, followed by resuspension in 2X SDS sample buffer, samples were heated at 95°C for 5 min and resolved by 10% SDS–PAGE and subjected to transfer into the nitrocellulose membranes. Membranes were blocked with 5% non-fat dry milk in PBS plus 0.1% Tween 20 (PBST) buffer for 1 hour at room temperature. Membranes were washed with PBST three times and then incubated with appropriate primary antibodies for 24 hours at 4°C. Total ERK 2 and Orai-1 (Santa Cruz Biotechnology), calmodulin (Abcam), AKAP79 (BD Transduction Laboratories), Calcineurin A subunit and GFP (Cell signaling) primary antibodies were used at dilutions of 1:5000 (ERK2), 1: 1000 (orai-1, calmodulin, AKAP79, CaN and GFP). The membranes were then washed with PBST again and incubated with 1:2500 dilutions of peroxidase-linked anti-rabbit (Santa Cruz Biotechnology) or anti mouse IgG (BD Bioscience) for 1 hour at room temperature. After washing with PBST, the bands were detected by an enhanced chemiluminescence ECL-plus

Western blotting detection system (GE Healthcare). Blots were analyzed by UN-Scan IT software.

***Mass spectroscopy-***In-gel trypsin digestion: Gel bands of interest were excised after Coomassie blue staining and cut into 1 – 2 $mm^3$ gel pieces, which were placed into 1.5 mL sample tubes. Gel pieces were rinsed twice with wash solution for 18h in total (200 µL, 50% methanol, 5% acetic acid). The solutions were removed and gel pieces were dehydrated in acetonitrile (200 µL, 5 min). Supernatant were removed and gel pieces were dried in a vacuum centrifuge for 3 min. Disulfide reduction was performed with 10 mM DTT (30 µL) for 0.5 h, followed by alkylation with 100 mM iodoacetamide (30 µL) for 0.5 h. Supernatants were removed from the gel samples and dehydration with acetonitrile and evaporation performed as described above. Gel pieces were washed with 100 mM ammonium bicarbonate (200 µL, 10 min). Supernatants were removed and dehydration performed with acetonitrile and evaporation as above. The gel samples were then rehydrated on ice with freshly prepared trypsin solution (30 µL, 20 ng/µL sequencing grade trypsin [Promega] in 50 mM ammonium bicarbonate). After rehydration excess trypsin solution was removed and 50 mM ammonium bicarbonate (10 µL) was added to prevent dehydration of gel pieces. Gel samples were digested at 37ºC for 18h. The gel pieces were then extracted sequentially with 50mM ammonium bicarbonate (60 µL), 50% acetonitrile, 5% formic acid (60 µL) and 85% acetonitrile, 5% formic acid (60 µL). The combined extracts were evaporated in a vacuum centrifuge and were redissolved in 5% acetonitrile, 0.1% formic acid (20 µL) on an ultrasonic bath and transferred into LC-MS sample vials.

LC-MS/MS analysis-For the analysis of in-gel digested protein material, liquid chromatography was performed using an Ultimate 3000 nano-HPLC system (Dionex, Sunnyvale, CA, USA) comprising a WPS-3000 micro auto sampler, a FLM-3000 flow manager and

column compartment, a UVD-3000 UV detector, an LPG-3600 dual-gradient micro-pump, and an SRD-3600 solvent rack controlled by Hystar (Bruker Daltonics, Billerica, MA, USA) and DCMS link 2.0 software. Samples were concentrated on a trapping column Dionex (Sunnyvale, CA, USA), 300 µm i.d., 0.1 cm) at a flow rate of 20 µL/min. For the separation with a C18 Pepmap column (75 µm i.d., 15 cm, Dionex), a flow rate of 250 nL/min was used as generated by a cap-flow splitter cartridge (1/1000). Peptides were eluted by the application of a 30 min multi-step gradient using solvents A (98% $H_2O$, 2% acetonitrile, 0.1% formic acid) and B (80% acetonitrile, 20% water, 0.1% formic acid):

| Composition (% solvent B) | Run time (min) |
|---|---|
| 2-10 | 0-3 |
| 10-25 | 3-18 |
| 25-50 | 18-30 |
| 50-90 | 30-30.2 |

The liquid chromatography was interfaced directly with a 3D high capacity ion trap mass spectrometer (amaZon; Bruker Daltonics) utilizing 10 µm i.d. distal coated SilicaTips (New Objective, Woburn, MA, USA) and nano-ESI mode. SPS parameter settings on the ion trap were tuned for a target mass of 850 *m/z*, compound stability 100% and a smart ICC target of 250,000. MS/MS analysis was initiated on a contact closure signal triggered by HyStar software (version 3.2). Up to five precursor ions were selected per cycle with active exclusion (0.5 min) in collision-induced dissociation (CID) mode. CID fragmentation was achieved using helium gas and a 30%–200% collision energy sweep with amplitude 1.0 (ions are ejected from the trap as soon as they fragment).

Data processing and database searching-Raw LC-MS/MS data were processed and Mascot compatible files were created using

DataAnalysis 4.0 software (Bruker Daltonics). Database searches were performed using the Mascot algorithm (version 2.4) and the UniProt_SwissProt database with mammalian taxonomy restriction (v2012.09.17, number of entries 537,505, after taxonomy filter: 66,032). The following parameters were applied: 2+, 3+ and 4+ ions, peptide mass tolerance 0.3 Da, $^{13}C$ = 2, fragment mass tolerance 0.6 Da, number of missed cleavages: two, instrument type: ESI-TRAP, fixed modifications: Carbamidomethylation (Cys), variable modifications: Oxidation (Met).

**Statistics**-Results are presented as means ± S.E.M. Statistical significance was assessed using Student's t test for comparison between two groups or analysis of variance (ANOVA) followed by a *post hoc* Newman Keuls multiple comparison test for the difference between groups and considered significant at $p < 0.05$ (*); $p<0.01$ (**).

## SUPPLEMENTAL REFERENCES


[S1]. Kar P, Bakowski D, Di Capite J, Nelson C, & Parekh AB (2012) Different agonists recruit different stromal interaction molecule proteins to support cytoplasmic Ca2+ oscillations and gene expression. *Proceedings of the National Academy of Sciences USA* 109:6969-6974.

[S2]. Bakowski D, Glitsch MD, & Parekh AB (2001) An examination of the secretion-like coupling model for the activation of the Ca2+ release-activated Ca2+ current ICRAC in RBL-1 cells. *Journal of Physiology (Lond.)* 532:55-71.

[S3]. Singaravelu K*, et al.* (2011) Mitofusin 2 regulates STIM1 migration from the Ca2+ store to the plasma membrane in cells with depolarised mitochondria. *Journal of Biological Chemistry* 286:12189-12201.

[S4]. Kar P, Nelson C, & Parekh AB (2011) Selective activation of the transcription factor NFAT1 by calcium microdomains near Ca2+ release-activated Ca2+ (CRAC) channels. *Journal of Biological Chemistry* 286:14795-14803.

[S5]. Mullins FM, Park CY, Dolmetsch RE, & Lewis RS (2009) STIM1 and calmodulin interact with Orai1 to induce Ca2+-dependent inactivation of CRAC channels. *Proceedings of the National Academy of Sciences USA* 106:15495-15500.
