# Supplementary material for: Dynamic Assembly of a Membrane Signaling Complex Enables Selective Activation of NFAT by Orai1
Source: Curr Biol. 2014 Jun 16;24(12):1361–8. doi: 10.1016/j.cub.2014.04.046 (PMC4062936; doi:10.1016/j.cub.2014.04.046)
Supplement: Document S2. Article plus Supplemental Information [file mmc2.pdf]

Current Biology 24, 1361–1368, June 16, 2014 ©2014 The Authors http://dx.doi.org/10.1016/j.cub.2014.04.046

Report

Dynamic Assembly of a Membrane Signaling Complex Enables Selective Activation of NFAT by Orai1

Pulak Kar,[1] Krishna Samanta,[1] Holger Kramer,[1] Otto Morris,[1] Daniel Bakowski,[1] and Anant B. Parekh[1,*]
[1]Department of Physiology, Anatomy, and Genetics, Oxford University, Parks Road, Oxford OX1 3PT, UK

Summary

NFAT-dependent gene expression is essential for the development and function of the nervous, immune, and cardiovascular systems and kidney, bone, and skeletal muscle [1]. Most NFAT protein resides in the cytoplasm because of extensive phosphorylation, which masks a nuclear localization sequence. Dephosphorylation by the Ca^{2+}-calmodulin-activated protein phosphatase calcineurin triggers NFAT migration into the nucleus [2, 3]. In some cell types, NFAT can be activated by Ca^{2+} nanodomains near open store-operated Orai1 and voltage-gated Ca^{2+} channels in the plasma membrane [4, 5]. How local Ca^{2+} near Orai1 is detected and whether other Orai channels utilize a similar mechanism remain unclear. Here, we report that the paralog Orai3 fails to activate NFAT. Orai1 is effective in activating gene expression via Ca^{2+} nanodomains because it participates in a membrane-delimited signaling complex that forms after store depletion and brings calcineurin, via the scaffolding protein AKAP79, to calmodulin tethered to Orai1. By contrast, Orai3 interacts less well with AKAP79 after store depletion, rendering it ineffective in activating NFAT. A channel chimera of Orai3 with the N terminus of Orai1 was able to couple local Ca^{2+} entry to NFAT activation, identifying the N-terminal domain of Orai1 as central to Ca^{2+} nanodomain-transcription coupling. The formation of a store-dependent signaling complex at the plasma membrane provides for selective activation of a fundamental downstream response by Orai1.

Results and Discussion

Store-operated Ca^{2+} channels are a major conduit for Ca^{2+} influx in nonexcitable cells [6, 7]. The best characterized and most widely distributed store-operated channel is the Ca^{2+} release-activated Ca^{2+} (CRAC) channel [8, 9]. CRAC channels are activated following the loss of Ca^{2+} from the ER. The molecular basis of this process is becoming clear: store depletion leads to oligomerization of the ER protein STIM1, and the oligomers then migrate to ER-plasma membrane junctions, where they bind to the pore-forming subunit of the CRAC channel Orai1 and trigger the channel to open [10]. The crystal structure of *Drosophila* Orai1 reveals the channel to be a hexamer [11].

Local Ca^{2+} signals in the vicinity of open CRAC channels activate important cytoplasmic signaling molecules, including enzymes [12, 13], ion channels [14], and vesicular fusion proteins [15]. In all of these cases, the Ca^{2+}-dependent target is closely apposed to the Ca^{2+} channel, rapidly transducing the local Ca^{2+} signal into a physiological output. A more complex scenario arises when the activated target is located at a distance well beyond the realm of the CRAC channel Ca^{2+} microdomain, typically <10–20 nm in spatial extent [16]. This spatial disconnect is seen with certain cytoplasmic enzymes [17] and intracellular transcription factors, including c-*fos* [18, 19] and NFAT [20]. The majority of NFAT protein is retained within the cytoplasm through extensive phosphorylation [1], and dephosphorylation by calcineurin results in migration of the transcription factor into the nucleus. An important but unresolved question is how Ca^{2+} nanodomains near CRAC channels are sensed and relayed to cytoplasmic targets such as NFAT.

Bioinformatic analysis and site-directed mutagenesis studies have identified a calmodulin-binding domain on the N terminus of Orai1, between residues 68 and 90 [21]. Specific single point mutations within this domain alter calmodulin binding to Orai1, without affecting the activation of Orai1 channels [21]. We therefore examined the effects of these mutations on NFAT activation following CRAC channel opening. Transfection into HEK293 cells of either Orai1 or Orai1 constructs containing point mutations within the N terminus of Orai1 that suppressed calmodulin binding (A73E, W76A) [21] together with STIM1 resulted in robust store-operated Ca^{2+} entry following store depletion with the SERCA pump blocker thapsigargin (Figure 1A), and no differences in either Ca^{2+} release or Ca^{2+} entry rates were seen with the different constructs (Figure 1A). To measure NFAT activation, we cotransfected cells with an NFAT1(1-460)-GFP fusion protein [4], STIM1, and either wild-type Orai1 or one of the two mutant Orai1 constructs. Whereas robust NFAT migration into the nucleus occurred after stimulation with thapsigargin in cells transfected with wild-type Orai1 (Figures 1B and 1F), significantly less NFAT activation occurred in the presence of A73E Orai1 (Figures 1C and 1F) or W76A Orai1 (Figures 1D and 1F). We measured coupling between CRAC channels and gene expression through use of a reporter gene (GFP) driven by an NFAT promoter [4, 20, 22]. Stimulation of RBL-1 cells (transfected with Orai1, STIM1, and reporter gene) with the physiological trigger leukotriene C_4, acting on cysteinyl leukotriene type I receptors, triggered GFP expression in ~30% of the cells, and this was significantly reduced when A73E or W76A Orai1 was expressed instead (Figures 1G and 1H; data are normalized to cells transfected with nonmutated Orai1). These results suggest that mutations within the calmodulin-binding domain of Orai1 interfere with NFAT activation and subsequent gene expression. Mutation of a tyrosine residue to alanine (Y80A) in Orai1 revealed strong calmodulin association with the channel [21]. NFAT-GFP migration into the nucleus was reduced following stimulation with thapsigargin in cells cotransfected with Orai1Y80A and STIM1 (Figures 1E and 1F), as was gene expression (Figure 1H), despite the cytoplasmic Ca^{2+} signals being unaffected (Figure 1A). Further discussion of this mutant is presented below.

Calmodulin should be located close to the Orai1 channel pore if it is to detect the Ca^{2+} nanodomain near each open CRAC channel. Two arguments suggest that this is the case.

*Correspondence: anant.parekh@dpag.ox.ac.uk
This is an open access article under the CC BY license (http://creativecommons.org/licenses/by/3.0/).

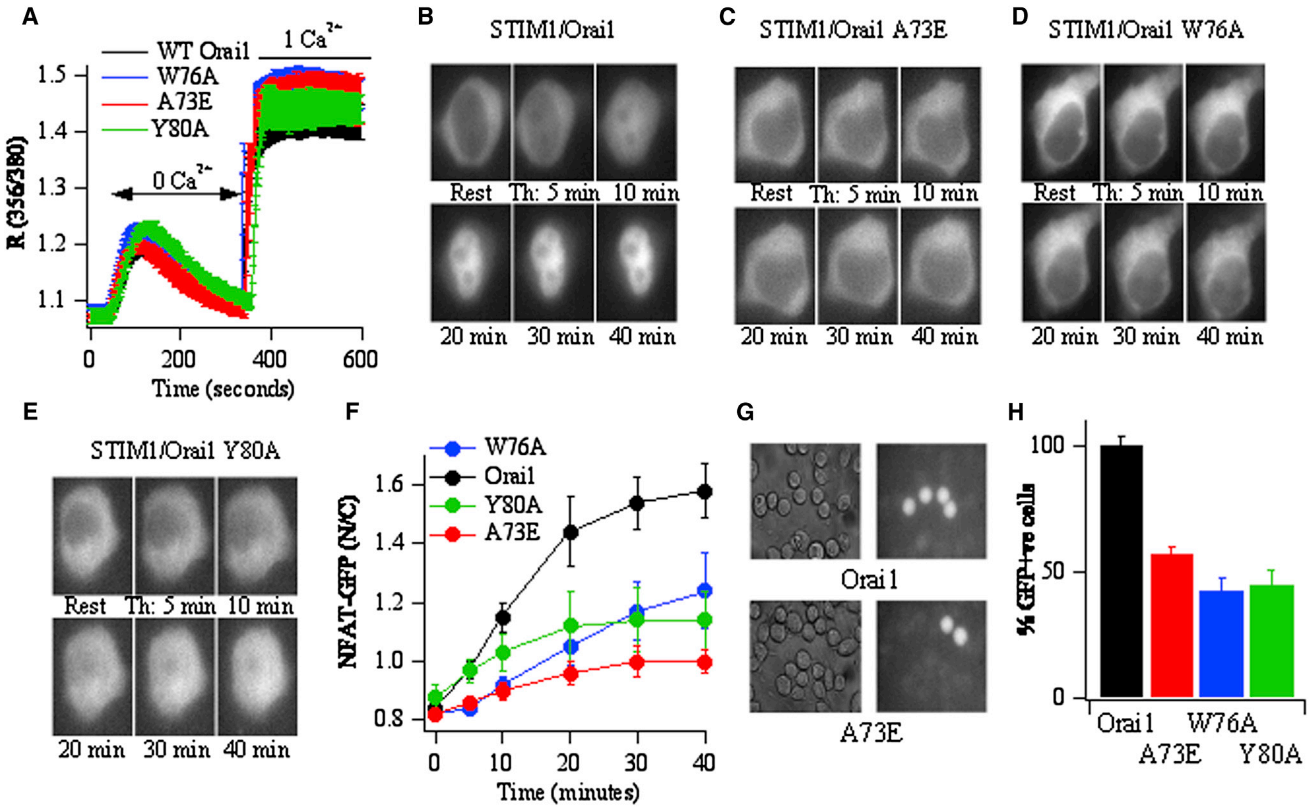

Figure 1. Mutations in the Calmodulin-Binding Domain of Orai1 Impair NFAT Activation

(A) Ca^{2+} release and subsequent store-operated Ca^{2+} entry to thapsigargin (2 μM) are shown in HEK293 cells transfected with STIM1 together with either nonmutated (WT) Orai1, A73E Orai1, W76A Orai1, or Y80A Orai1. Each trace is the average of between 40 and 65 cells.

(B) Images show movement of NFAT-GFP from cytosol to nucleus in an individual HEK293 cell transfected with STIM1 and Orai1 following continuous thapsigargin stimulation in 2 mM external Ca^{2+}. Time in thapsigargin is indicated below each image.

(C–E) Identical protocol to (B), but cells have been transfected with STIM1 and A73E Orai1 (C), STIM1 and W76A Orai1 (D), or STIM1 and Y80A Orai1 (E).

(F) Graph comparing kinetics of NFAT-GFP movement into the nucleus (depicted as nuclear/cytosolic ratio) for the different conditions. Each trace is the average of between 11 and 15 individual cells.

(G) Reporter gene expression in response to 160 nM LTC_4, compared between RBL-1 cells transfected with Orai1 or A73E Orai1. STIM1 and GFP under an NFAT promoter were cotransfected in both cases. Left panels show transillumination images.

(H) Aggregate data from five independent experiments. Data have been normalized to the percentage of cells expressing a reporter gene in Orai1-transfected cells.

Error bars show SEM.

First, Ca^{2+}-dependent fast inactivation of CRAC channels, which is thought to be mediated by calmodulin [21, 23], is reduced by loading the cytoplasm with the fast Ca^{2+} chelator BAPTA, but not the slower EGTA, placing the Ca^{2+} binding site within ~7 nm of the pore [24–26]. Consideration of the voltage dependence of fast inactivation combined with simulation of the local Ca^{2+} signal near the open channel puts the calmodulin site at <5.4 nm from the pore (see Figure S1 available online and associated text for details). Second, if calmodulin is indeed located close to the pore, then the rate and extent of fast inactivation should correlate with the speed of buildup and the size of the Ca^{2+} nanodomain, and this was indeed the case (Figure S1 and corresponding text).

When bound to the isoleucine-glutamine (IQ) domain of voltage-gated Ca^{2+} channels, calmodulin is shielded from pharmacological blockers [27]. If calmodulin is tethered to Orai1 or a closely related protein, we reasoned that it should also be relatively insensitive to such inhibitors. Consistent with this, NFAT migration into the nucleus was unaffected by the calmodulin inhibitor calmidazolium (Figures 2A and 2B). Following stimulation with thapsigargin in Ca^{2+}-free solution, the decay of the Ca^{2+} signal is due mainly to the plasma membrane Ca^{2+}ATPase pump [28], which is stimulated by calmodulin. The rate of Ca^{2+} clearance was slowed by calmidazolium (Figure 2C), confirming that calmidazolium was able to inhibit calmodulin in these cells. Does Ca^{2+} entry through Orai1 release a fraction of calmodulin from the channel? To test this, we carried out several independent experiments. First, we immunoprecipitated full-length Orai1-GFP and then blotted for endogenous calmodulin. Whereas strong interaction was found when the lysis buffer contained low Ca^{2+} (4 mM EGTA), the association between the proteins was significantly reduced, although not abolished, in high Ca^{2+} (Figures S2A and S2B). Conversely, after immunoprecipitation of calmodulin-GFP, we found significantly more association of endogenous Orai1 in low-Ca^{2+}, but not high-Ca^{2+}, lysis buffer (Figures S2C and S2D). We validated the use of the Orai1 antibody in the following manner. Knockdown of Orai1 using a small interfering RNA (siRNA) approach reduced CRAC channel activity by ~70% [29] and reduced Orai1 expression by 62% ± 7% (Figure S2E). Hence, the anti-Orai1 antibody recognizes the protein.

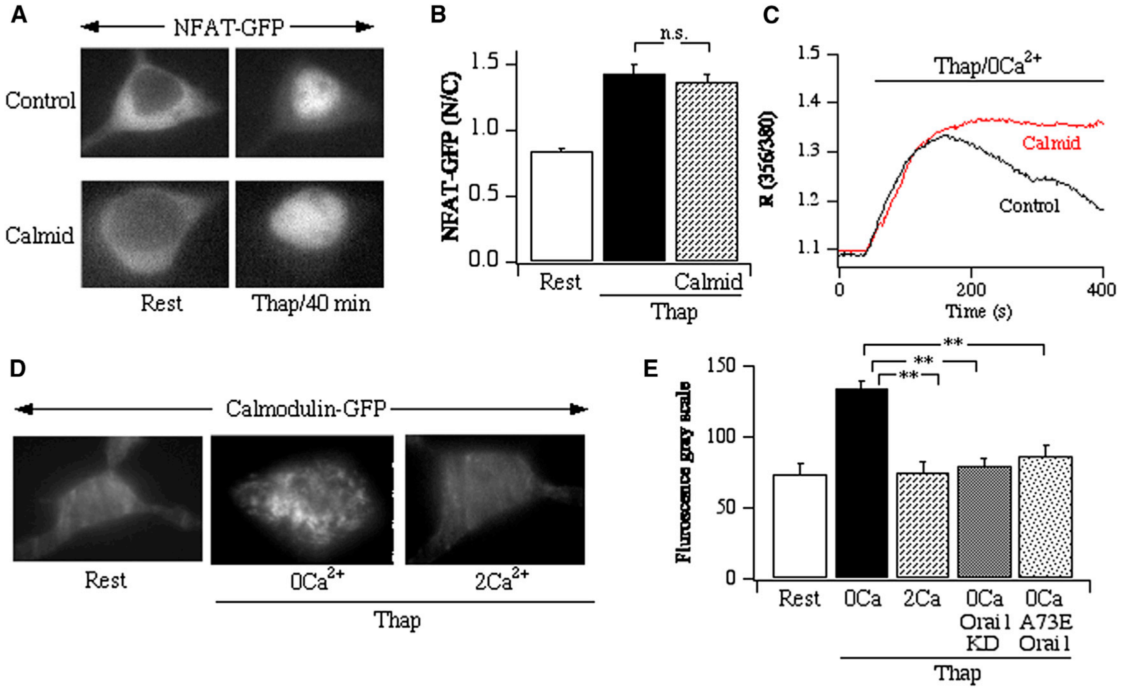

Figure 2. Calmodulin and Orai1 Interact in a Ca^{2+}-Dependent Manner

(A) NFAT activation occurs in the presence of calmidazolium (10 μM; 15 min pretreatment).

(B) Aggregate data are summarized (rest, 21 cells; thapsigargin, 11 cells; calmidazolium + thapsigargin, 10 cells).

(C) Calmidazolium (10 μM; 15 min pretreatment) slows Ca^{2+} extrusion by the Ca^{2+}-calmodulin-dependent plasma membrane Ca^{2+}ATPase pump. Cells were stimulated with thapsigargin in Ca^{2+}-free solution. The decay of the Ca^{2+} signal is mainly due to Ca^{2+}ATPase pump activity.

(D) TIRF microscopy images following overexpression of nontagged Orai1, STIM1, and calmodulin-GFP.

(E) Aggregate data plots of total GFP fluorescence in the evanescent field from several experiments as in (D). Each histogram is the mean of 10–13 cells (three preparations).

Error bars show SEM. n.s., not significant; $^{**}p < 0.01$.

We used tandem mass spectroscopy to confirm the identity of the gel band in low-Ca^{2+} buffer. Pull-down of calmodulin-GFP revealed the presence of Orai1 in lysis buffer containing 4 mM EGTA (Figure S2F), confirming the presence of both proteins in the gel band in low Ca^{2+}. Following transfection with GFP alone, immunoprecipitation of GFP failed to reveal the presence of calmodulin in either 4 mM EGTA or 2 mM $CaCl_2$ (Figure S2G), ruling out interaction between GFP and calmodulin. In a further set of experiments, we used total internal reflection fluorescence (TIRF) microscopy to measure the subplasmalemmal distribution of calmodulin before and then after store depletion. Coexpression of calmodulin-GFP and Orai1 (and STIM1) resulted in a general smearing of fluorescence within the evanescent field at rest (Figure 2D). Store depletion in the absence of external Ca^{2+} led to the formation of puncta of calmodulin-GFP, which resembled those formed by STIM1/Orai1 after store depletion [29]. Total GFP fluorescence in the evanescent field increased slightly after store depletion, suggesting modest recruitment of calmodulin-GFP along with redistribution within the field. Readmission of external Ca^{2+} led to a reduction in calmodulin-GFP puncta (Figure 2D; aggregate data are summarized in Figure 2E). Puncta were qualitatively less prominent after transfection with calmodulin-GFP alone (data not shown) or after knockdown of endogenous Orai1 (Figure 2E). Transfection of calmodulin-GFP, STIM1, and A73EOrai1 also resulted in fewer puncta (Figure 2E). Following expression of calmodulin-GFP, STIM1, and Orai1-cherry, we observed punctate-like structures of calmodulin-GFP and Orai1-cherry after store depletion in the absence of external Ca^{2+}, which colocalized well, at least within the limit of resolution of confocal microscopy (Figure S3A). Addition of external Ca^{2+} disassembled the calmodulin-GFP puncta without affecting cherry-Orai1 clusters (Figure S3A). Studies with a myc-tagged Ca^{2+}-insensitive mutant calmodulin protein (in which all four EF hands had been mutated [30]) provided further evidence in support of interaction between Orai1 and calmodulin (Figure S2H). This mutant protein is Ca^{2+} insensitive and therefore should remain associated with Orai1 even in high Ca^{2+}. Following expression of Orai1-GFP and myc-tagged mutant calmodulin, immunoprecipitation of GFP revealed the presence of mutant calmodulin in both the absence and presence of Ca^{2+}. Collectively, these results are consistent with the view that calmodulin is tethered close to Orai1 at low resting cytosolic Ca^{2+} levels and that a fraction is released from the channel following Ca^{2+} entry through CRAC channels.

Although our studies show Ca^{2+}-independent association between Orai1 and calmodulin in low Ca^{2+}, a previous study did not observe such an interaction [21]. We do not have an explanation for this, but subtle differences in experimental conditions might contribute. In addition, we cannot rule out an indirect association between calmodulin and Orai1, mediated through a bridging protein. Our observation of an apocalmodulin site on Orai1 or a closely associated protein would be consistent both with the kinetics of Ca^{2+}-calmodulin-dependent fast inactivation of CRAC channels (which develops

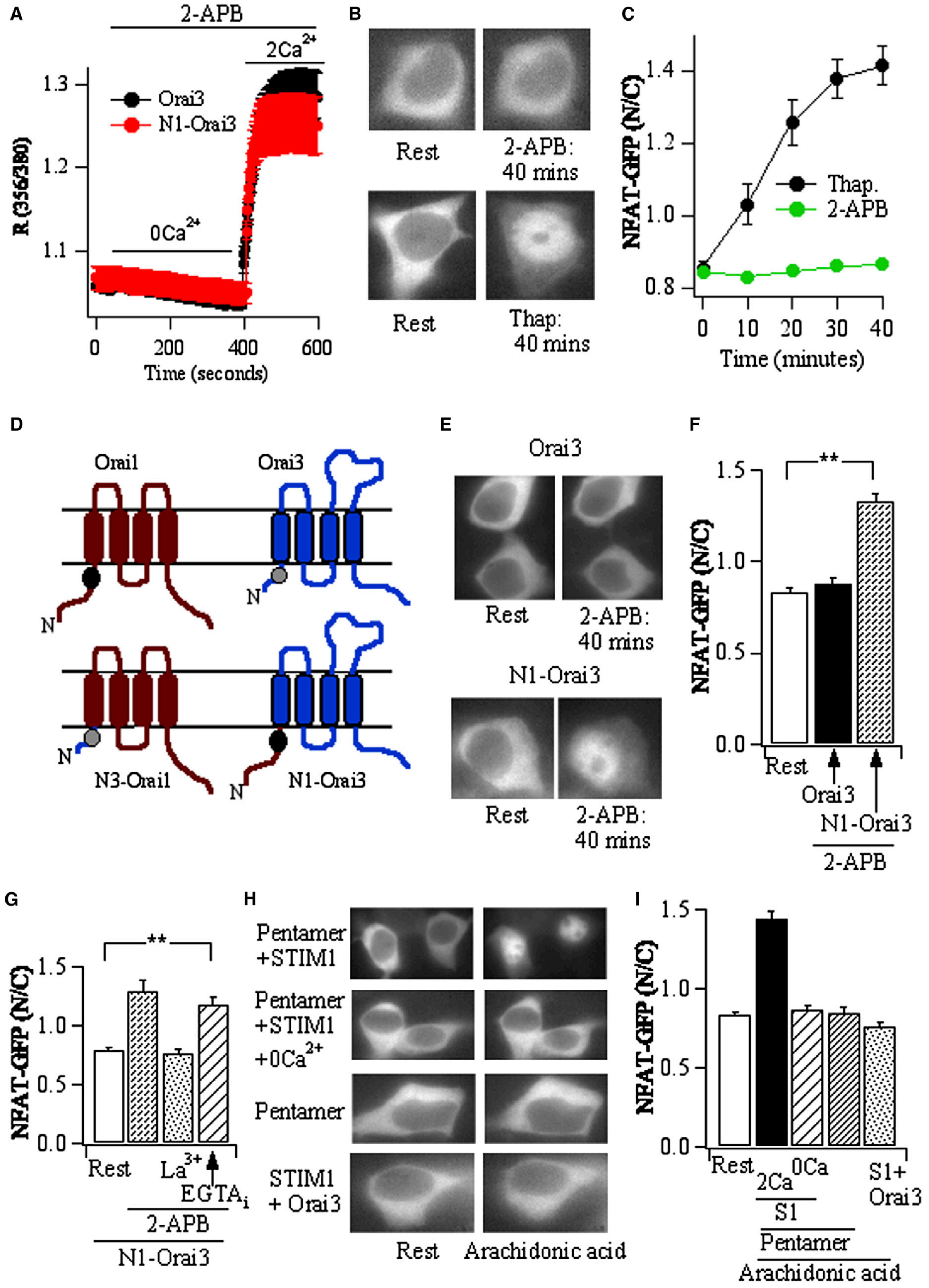

Figure 3. The N Terminus of Orai1 Couples Local Ca^{2+} to NFAT Activation

(A) Following transfection with Orai3 and STIM1, acute exposure to 50 μM 2-APB evokes a large cytoplasmic Ca^{2+} rise (>50 cells). The N1-O3 construct also elicited a similar Ca^{2+} signal.

(legend continued on next page)

rapidly with an initial time constant of 10 ms) and with the observation that the mutant Ca^{2+}-insensitive calmodulin protein reduces fast inactivation [23] (Figure S1).

We asked whether the ability of Orai1 to activate NFAT was unique to the channel, or whether other store-operated Ca^{2+} channels could also recruit this pathway. Orai3 is a paralog of Orai1 and functions as a store-operated Ca^{2+} channel in expression systems [31–34]. Orai3 is gated directly by the pharmacological agent 2-aminoethoxydiphenyl borate (2-APB), which binds to the protein and dilates the channel pore [35–38]. Robust cytoplasmic Ca^{2+} signals were evoked when Ca^{2+} was applied to 2-APB-treated HEK293 cells coexpressing Orai3 and STIM1 (Figure 3A), and these were substantially smaller in nontransfected cells (data not shown). The Ca^{2+} signal to 2-APB in Orai3-expressing cells was similar to that evoked by store depletion in Orai1-expressing cells (Figure 1A), yet the former consistently failed to activate NFAT (Figures 3B and 3C). This was not due to an inhibitory effect of 2-APB on the downstream signaling pathway, because raising bulk cytoplasmic Ca^{2+} with a high concentration of ionomycin caused robust NFAT activation after exposure to 2-APB (Figure S3B). When gated by 2-APB, Orai3 channels are permeable to both Na^{+} and Ca^{2+} [37, 38]. Na^{+} flux through the channels would depolarize the membrane potential, thus reducing the size and radial spread of the local Ca^{2+} signal. To circumvent this, we applied 2-APB in low-Na^{+} (10 mM) external solution. However, NFAT still did not migrate to the nucleus (Figure 3E, upper panel). Although sequence alignment revealed several conserved residues within the N terminus calmodulin-binding domain of Orai1 with a corresponding stretch in Orai3, the N terminus of Orai3 is considerably shorter than Orai1. We reasoned that a calmodulin-binding domain in Orai3 [39], if functional, would be less efficacious than Orai1 in coupling local Ca^{2+} signals to NFAT. We therefore made two chimeras: in one, we replaced the N terminus of Orai1 with that from Orai3 (N3-Orai1; Figure 3D), and in the other, we replaced the N terminus of Orai3 with that from Orai1 (N1-Orai3; Figure 3D). The N3-Orai1 construct failed to produce a clear Ca^{2+} signal following stimulation with thapsigargin, consistent with a previous report of poor functional expression [40]. By contrast, N1-Orai3 channels responded to 2-APB by generating detectable Ca^{2+} signals (Figure 3A). Whereas 2-APB failed to stimulate NFAT movement in those cells transfected with Orai3 channels (Figures 3E and 3F), NFAT activation occurred when N1-Orai3 channels were expressed instead (Figures 3E and 3F), although fewer transfected cells responded (~2-fold less) compared with stimulation with thapsigargin in cells overexpressing STIM1 and Orai1. NFAT activation to 2-APB in N1-Orai3-expressing cells was suppressed by omission of external Ca^{2+} or by block of the Orai3 channels with La^{3+} (Figure 3G) but was unaffected by loading the cytoplasm with the slow Ca^{2+} chelator EGTA (Figure 3G). The presence of the N terminus of Orai1 therefore helps enable Orai3 to activate cytoplasmic NFAT via local Ca^{2+} entry.

Fast inactivation of CRAC channels is thought to require Ca^{2+}-dependent binding of calmodulin to the N terminus of Orai1 [21]. Tethered calmodulin also appears necessary for activation of NFAT, and this would require Ca^{2+}-dependent dissociation from Orai1, presumably from the apocalmodulin site. CaV1.3 channels have two distinct calmodulin-binding sites: a vestigial EF hand region upstream of the IQ domain on the C terminus, and an N-terminal spatial Ca^{2+} transforming element module. Ca^{2+}-calmodulin is able to shuttle between the two, with different effects on channel activity [41]. It is conceivable that there are two calmodulin-binding sites on the N terminus of Orai1 and that mutations in the Ca^{2+}-calmodulin binding site (e.g., A73) destabilize the apocalmodulin region. Alternatively, specific mutations with the calmodulin-binding domain might result in weak association between Orai1 and a bridging protein that brings calmodulin close to the Orai1 pore. In the Y80A Orai1 mutant, calmodulin remains bound in both low and high Ca^{2+} (Figure S2I), and this is associated with strong, fast inactivation of the channel. The weaker activation of NFAT with this construct could therefore reflect a slower dissociation rate of calmodulin from Orai1 and/or the reduction in local Ca^{2+} influx following opening of the mutant channel [21].

Can the presence of Orai1 within a heteromultimeric channel complex (containing Orai1 and non-Orai1 components) confer the ability to couple local Ca^{2+} to NFAT activation, when the other subunits within the multimer are ineffective? To test this, we transfected cells with an arachidonic acid-gated non-store-operated Ca^{2+} channel, a pentamer of three Orai1 and two Orai3 subunits, along with the regulator STIM1 [33]. Stimulation with arachidonic acid resulted in robust NFAT migration into the nucleus (Figures 3H and 3I). No detectable movement occurred in the absence of external Ca^{2+} or when the pentamer was expressed without STIM1 (Figures 3H and 3I). Arachidonic acid failed to stimulate NFAT movement in cells cotransfected with STIM1 and Orai3 (Figures 3H and 3I) or with STIM1 and Orai1 (zero of six cells showed movement). Inclusion of Orai1 in a heteromeric channel complex thus imparts the ability to transduce local Ca^{2+} signals into NFAT activation. We asked whether the preferential coupling of Orai1 to NFAT was due exclusively to tethered calmodulin.

(B) Despite eliciting robust Ca^{2+} entry, 2-APB fails to drive NFAT migration into the nucleus (upper panels). By contrast, stimulation with thapsigargin evokes clear NFAT movement in control cells from the same preparations.

(C) Aggregate data plotting nuclear/cytosolic NFAT versus time, compared between cells activated with thapsigargin (11 cells, mock transfected and from the same preparations as those used for 2-APB) and cells activated with 2-APB (17 cells, transfected with STIM1 and Orai3).

(D) Cartoon depicting chimeras that were synthesized. The dark, filled circle on Orai1 is calmodulin. It has been presented as less dark on Orai3, since the interaction appears weaker.

(E) Whereas 2-APB fails to activate NFAT movement in cells expressing Orai3, it caused clear movement in a fraction of cells transfected with the N1-Orai3 construct. In these experiments, external Na^{+} was 10 mM (replaced with $TRIS^{+}$). Cells were stimulated in low-Na^{+} (10 mM) solution containing 2 mM Ca^{2+}.

(F) Aggregate data from experiments in (E). Each bar represents >12 cells.

(G) 2-APB-activated NFAT movement in N1-Orai3 cells is suppressed by the CRAC channel blocker La^{3+} (50 μM) but is unaffected by loading the cytoplasm with the slow Ca^{2+} chelator EGTA (five to nine cells for each condition).

(H) NFAT migrates into the nucleus in response to stimulation with 8 μM arachidonic acid in cells expressing the pentamer and STIM1 in the presence of external Ca^{2+}, but not when Ca^{2+} is absent or when STIM1 is not cotransfected. Arachidonic acid does not activate NFAT in cells transfected with Orai3 and STIM1.

(I) Aggregate data from experiments as in (H). Each bar is the average of 20–35 cells.

Error bars show SEM. **$p < 0.01$.

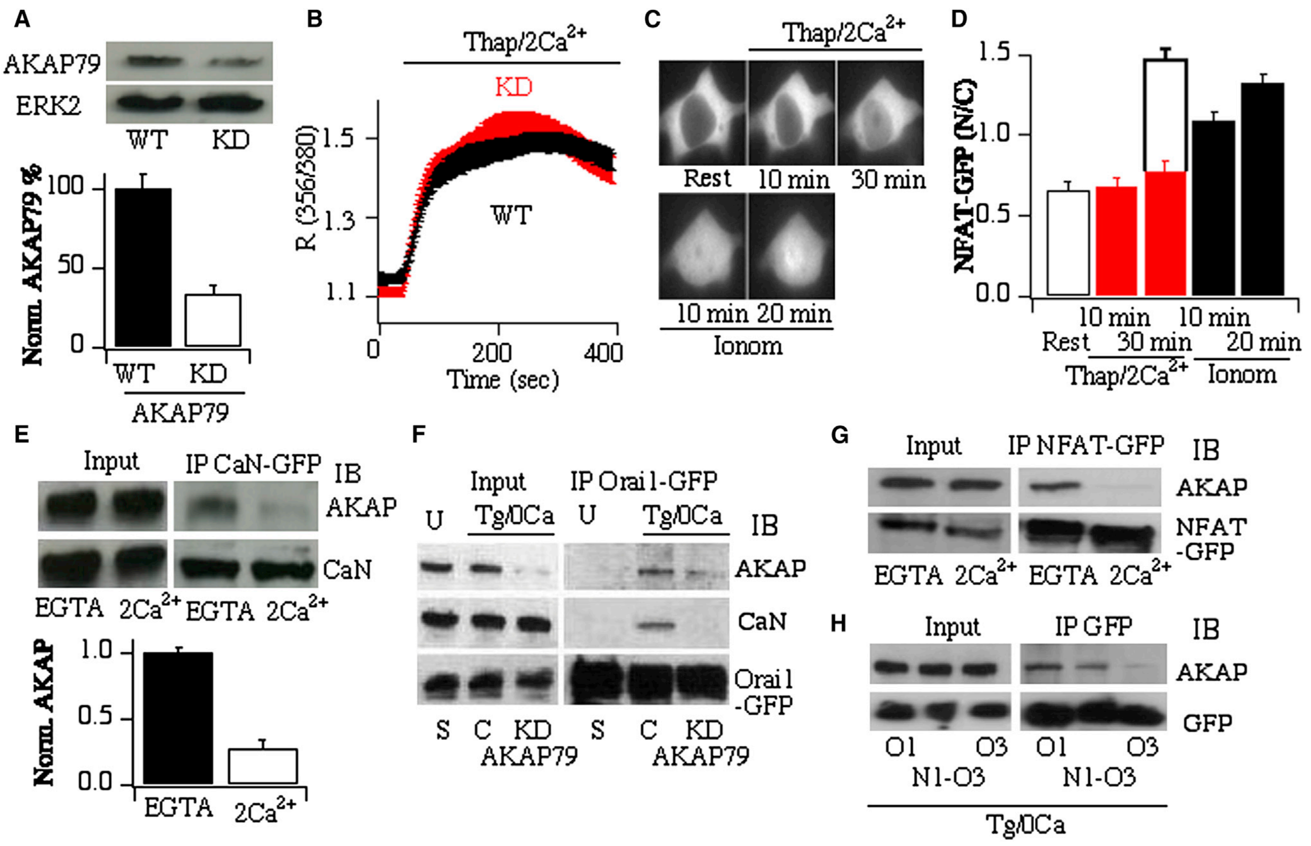

Figure 4. Formation of a Store-Dependent Signaling Complex

(A) Western blot showing that siRNA against AKAP79 reduces protein expression. KD, knockdown.

(B) Knockdown (KD) of AKAP79 fails to alter the Ca^{2+} signal to thapsigargin (2 μM) in external Ca^{2+} (average of >50 cells per condition).

(C) NFAT-GFP nuclear migration is reduced after knockdown of AKAP79. Subsequent stimulation with ionomycin (5 μM) in the same cell led to strong movement to the nucleus.

(D) Aggregate data from several experiments. Red bars denote cells treated with siRNA against AKAP79. The open bar at 30 min shows the response to thapsigargin in mock-transfected cells at the same time point. Black bars denote the response to ionomycin, applied 10 or 20 min after thapsigargin treatment for 30 min in cells treated with siRNA against AKAP79.

(E) Coimmunoprecipitation experiments show strong interaction between AKAP79 and calcineurin in low-Ca^{2+}, but not high-Ca^{2+}, lysis buffer. Calcineurin-GFP was pulled down, and immunoblot (IB) was for endogenous AKAP79.

(F) Immunoprecipitation of Orai1-GFP was associated with little calcineurin in unstimulated cells (labeled U), but the extent of interaction increased after store depletion in Ca^{2+}-free external solution. Interaction was reduced after knockdown of AKAP79. Cells were exposed to thapsigargin in Ca^{2+}-free solution for 5 min before lysis in 4 mM EGTA-containing buffer. S denotes transfection with scrambled siRNA; C represents control cells (stimulated with thapsigargin in Ca^{2+}-free solution) for the AKAP knockdown data set.

(G) Blots show association of NFAT-GFP with AKAP79 in low-Ca^{2+} (EGTA), but not high-Ca^{2+}, lysis buffer. In (G) and (H), gels are representative of three independent experiments.

(H) Pull-down experiments comparing extent of interaction between Orai1, N1-Orai3, and Orai3 channels (all GFP-tagged) and AKAP79.
Error bars show SEM.

Pull-down experiments revealed association of calmodulin with Orai3 (Figure S3C), but this was significantly less than that seen with Orai1. Although the N1-Orai3 protein had more associated calmodulin than Orai3, this was clearly less than for Orai1 (Figure S3C). We therefore reasoned that an additional mechanism contributed to effective coupling between Orai1 and NFAT.

Drawing on findings from voltage-gated Ca^{2+} channels, where calcineurin can be held close to CaV1.2 [42], we considered that calcineurin might bind to Orai1. Coimmunoprecipitation studies failed to reveal the presence of calcineurin when Orai1-GFP was pulled down in resting, untreated cells (Figure 4F, labeled U), arguing against association of calcineurin to Orai1 under these conditions. An elegant study in hippocampal neurons demonstrated that the scaffold protein AKAP79 recruited calcineurin to L-type channels [5]. We therefore explored the possibility that AKAP79 orchestrated the reversible formation of an Orai1-calcineurin complex following store depletion. Knockdown of AKAP79 using a siRNA approach resulted in ~65% reduction of protein expression (Figure 4A). The Ca^{2+} signal evoked by thapsigargin was unaffected by the decreased levels of AKAP79 (Figure 4B), but NFAT activation was significantly impaired (Figures 4C and 4D). Even 30 min after stimulation, little NFAT had migrated into the nucleus (Figure 4D, red bars; the open bar in Figure 4D at 30 min denotes NFAT movement in mock-transfected cells under similar conditions). High, nonphysiological levels of cytoplasmic Ca^{2+} activate NFAT independent of local Ca^{2+} entry in HEK cells [4]. NFAT migration was recovered in AKAP79-deficient cells following a large

elevation of bulk Ca^{2+} independent of Orai1 by stimulating cells with a high concentration of the Ca^{2+} ionophore ionomycin (Figure 4D). Therefore, loss of AKAP79 does not impair the ability of Ca^{2+} to recruit the NFAT pathway per se; it inhibits Orai1 coupling to NFAT. Pull-down of calcineurin-GFP revealed the presence of AKAP79 in low Ca^{2+} but significantly less association in high Ca^{2+} (Figure 4E). This could reflect Ca^{2+}-dependent reduction in calcineurin affinity for AKAP79 or a redistribution of the bound phosphatase to other substrates when Ca^{2+} is raised. Whereas pull-down of Orai1-GFP revealed little association of calcineurin in nonstimulated cells, interaction was revealed after store depletion with thapsigargin (Figure 4F). Knockdown of AKAP79 resulted in loss of interaction between Orai1 and calcineurin after store depletion (Figure 4F). AKAP79 therefore brings calcineurin close to Orai1, thereby juxtaposing the phosphatase with its activator calmodulin. We also found some association of NFAT-GFP with AKAP79 in resting cells, and this was reduced in elevated Ca^{2+} (Figure 4G). NFAT dephosphorylation occurred under these conditions, which might have resulted in dissociation or reorganization of a resting NFAT complex [43] and the loss of interaction with AKAP79. By contrast with Orai1, Orai3 failed to interact with AKAP79 (Figure 4H) or calcineurin (Figure S3D) to any detectable extent. The N1-Orai3 protein rescued, albeit partially, interaction between the channel and AKAP79 (Figure 4H).

Whereas overexpression of wild-type AKAP79 had no effect on either thapsigargin-evoked Ca^{2+} signals or on the ability of NFAT-GFP to migrate to the nucleus (Figures S4A–S4C), expression of an AKAP79 mutant (ΔPIX-AKAP79) that cannot bind calcineurin [42] inhibited NFAT accumulation in the nucleus without altering the Ca^{2+} signal (Figures S4A–S4C). Both proteins were expressed at similar levels (Figure S4D). Pull-down experiments confirmed that ΔPIX-AKAP79 failed to interact with calcineurin (Figure S4E). Furthermore, following knockdown of AKAP79, transfection with wild-type AKAP79, but not ΔPIX-AKAP79, rescued NFAT activation (Figures S4B and S4C).

A major question in Ca^{2+} signaling is how Ca^{2+} from different sources activates distinct cellular responses. In many cell types in various species, local Ca^{2+} signals near open Ca^{2+} channels selectively stimulate downstream targets, an effect often mediated through calmodulin. Although Ca^{2+} dissociates relatively slowly from the C-lobe of calmodulin, we calculate a diffusion distance of $\sim$0.5–0.8 μm before Ca^{2+} is released from the protein. This prompts the question: how does a downstream target located a few micrometers away from the plasma membrane distinguish Ca^{2+}-calmodulin activated by local Ca^{2+} near one class of Ca^{2+} channel from that generated by other channels or from the relatively large amounts of free calmodulin in the cytoplasm? And how does Ca^{2+}-calmodulin from one Ca^{2+} source activate one target and not others? Our findings shed new insight into this universal decoding problem by revealing the presence of a store-dependent mechanism that brings the activator (calmodulin) into close proximity to the intermediary (calcineurin) and executor (NFAT). Calmodulin is tethered to Orai1 or a closely associated protein and is therefore held close to the source of Ca^{2+}. This pool of calmodulin has privileged access to calcineurin because store depletion recruits AKAP79 with attached calcineurin to Orai1. This form of coincidence detection, requiring two conditions to be satisfied (tethered calmodulin and store-dependent association of AKAP79, the latter bringing calcineurin and NFAT to Orai1), provides a mechanism for ensuring that only local Ca^{2+} entry via store depletion will activate the pathway. Ca^{2+} release from stores or local Ca^{2+} entry through other channels such as Orai3 will not be effective because AKAP79 is not recruited to, and there is less calmodulin associated with, Orai3. This dual requirement for tethered calmodulin and AKAP in mast cells prevents physiological fluctuations in cytosolic Ca^{2+} from activating NFAT in the absence of store depletion. Large, nonphysiological bulk rises in Ca^{2+} can activate NFAT, even in the absence of AKAP79 (Figure 4D), by raising cytoplasmic Ca^{2+} sufficiently to match the local physiological Ca^{2+} rise near Orai1. Given that impaired calcineurin-NFAT signaling is linked to various disorders in nonexcitable cells, including autoimmune disease, osteoporosis, Down syndrome, and possibly cancer [1], the privileged pathway between Orai1 and NFAT identifies the channel as a potential new target for management of these diseases.

Supplemental Information

Supplemental Information includes four figures and Supplemental Experimental Procedures and can be found with this article online at http://dx.doi.org/10.1016/j.cub.2014.04.046.

Acknowledgments

This work was supported by an MRCUK grant to A.B.P. We thank Michael Berridge for helpful comments on the manuscript.

Received: May 20, 2013
Revised: April 4, 2014
Accepted: April 23, 2014
Published: June 5, 2014

References

1. Hogan, P.G., Chen, L., Nardone, J., and Rao, A. (2003). Transcriptional regulation by calcium, calcineurin, and NFAT. Genes Dev. *17*, 2205–2232.
2. Müller, M.R., and Rao, A. (2010). NFAT, immunity and cancer: a transcription factor comes of age. Nat. Rev. Immunol. *10*, 645–656.
3. Wu, H., Peisley, A., Graef, I.A., and Crabtree, G.R. (2007). NFAT signaling and the invention of vertebrates. Trends Cell Biol. *17*, 251–260.
4. Kar, P., Nelson, C., and Parekh, A.B. (2011). Selective activation of the transcription factor NFAT1 by calcium microdomains near Ca2+ release-activated Ca2+ (CRAC) channels. J. Biol. Chem. *286*, 14795–14803.
5. Li, H., Pink, M.D., Murphy, J.G., Stein, A., Dell'Acqua, M.L., and Hogan, P.G. (2012). Balanced interactions of calcineurin with AKAP79 regulate Ca2+-calcineurin-NFAT signaling. Nat. Struct. Mol. Biol. *19*, 337–345.
6. Parekh, A.B., and Putney, J.W.J., Jr. (2005). Store-operated calcium channels. Physiol. Rev. *85*, 757–810.
7. Hogan, P.G., Lewis, R.S., and Rao, A. (2010). Molecular basis of calcium signaling in lymphocytes: STIM and ORAI. Annu. Rev. Immunol. *28*, 491–533.
8. Hoth, M., and Penner, R. (1992). Depletion of intracellular calcium stores activates a calcium current in mast cells. Nature *355*, 353–356.
9. Parekh, A.B. (2010). Store-operated CRAC channels: function in health and disease. Nat. Rev. Drug Discov. *9*, 399–410.
10. Soboloff, J., Rothberg, B.S., Madesh, M., and Gill, D.L. (2012). STIM proteins: dynamic calcium signal transducers. Nat. Rev. Mol. Cell Biol. *13*, 549–565.
11. Hou, X., Pedi, L., Diver, M.M., and Long, S.B. (2012). Crystal structure of the calcium release-activated calcium channel Orai. Science *338*, 1308–1313.
12. Willoughby, D., Everett, K.L., Halls, M.L., Pacheco, J., Skroblin, P., Vaca, L., Klussmann, E., and Cooper, D.M. (2012). Direct binding between Orai1 and AC8 mediates dynamic interplay between Ca2+ and cAMP signaling. Sci. Signal. *5*, ra29.
13. Ng, S.-W., di Capite, J.L., Singaravelu, K., and Parekh, A.B. (2008). Sustained activation of the tyrosine kinase Syk by antigen in mast cells

requires local Ca2+ influx through Ca2+ release-activated Ca2+ channels. J. Biol. Chem. *283*, 31348–31355.

14. Lemonnier, L., Prevarskaya, N., Shuba, Y., Vanden Abeele, F., Nilius, B., Mazurier, J., and Skryma, R. (2002). Ca2+ modulation of volume-regulated anion channels: evidence for colocalization with store-operated channels. FASEB J. *16*, 222–224.
15. Cheng, K.T., Liu, X., Ong, H.L., Swaim, W., and Ambudkar, I.S. (2011). Local Ca^2+ entry via Orai1 regulates plasma membrane recruitment of TRPC1 and controls cytosolic Ca^2+ signals required for specific cell functions. PLoS Biol. *9*, e1001025.
16. Parekh, A.B. (2008). Ca2+ microdomains near plasma membrane Ca2+ channels: impact on cell function. J. Physiol. *586*, 3043–3054.
17. Chang, W.C., Di Capite, J., Singaravelu, K., Nelson, C., Halse, V., and Parekh, A.B. (2008). Local Ca2+ influx through Ca2+ release-activated Ca2+ (CRAC) channels stimulates production of an intracellular messenger and an intercellular pro-inflammatory signal. J. Biol. Chem. *283*, 4622–4631.
18. Ng, S.-W., Nelson, C., and Parekh, A.B. (2009). Coupling of Ca(2+) microdomains to spatially and temporally distinct cellular responses by the tyrosine kinase Syk. J. Biol. Chem. *284*, 24767–24772.
19. Di Capite, J., Ng, S.-W., and Parekh, A.B. (2009). Decoding of cytoplasmic Ca(2+) oscillations through the spatial signature drives gene expression. Curr. Biol. *19*, 853–858.
20. Kar, P., Nelson, C., and Parekh, A.B. (2012). CRAC channels drive digital activation and provide analog control and synergy to Ca(2+)-dependent gene regulation. Curr. Biol. *22*, 242–247.
21. Mullins, F.M., Park, C.Y., Dolmetsch, R.E., and Lewis, R.S. (2009). STIM1 and calmodulin interact with Orai1 to induce Ca2+-dependent inactivation of CRAC channels. Proc. Natl. Acad. Sci. USA *106*, 15495–15500.
22. Kim, M.-S., and Usachev, Y.M. (2009). Mitochondrial Ca2+ cycling facilitates activation of the transcription factor NFAT in sensory neurons. J. Neurosci. *29*, 12101–12114.
23. Litjens, T., Harland, M.L., Roberts, M.L., Barritt, G.J., and Rychkov, G.Y. (2004). Fast Ca(2+)-dependent inactivation of the store-operated Ca2+ current (ISOC) in liver cells: a role for calmodulin. J. Physiol. *558*, 85–97.
24. Zweifach, A., and Lewis, R.S. (1995). Slow calcium-dependent inactivation of depletion-activated calcium current. Store-dependent and -independent mechanisms. J. Biol. Chem. *270*, 14445–14451.
25. Fierro, L., and Parekh, A.B. (1999). Fast calcium-dependent inactivation of calcium release-activated calcium current (CRAC) in RBL-1 cells. J. Membr. Biol. *168*, 9–17.
26. Hoth, M., and Penner, R. (1993). Calcium release-activated calcium current in rat mast cells. J. Physiol. *465*, 359–386.
27. Zühlke, R.D., Pitt, G.S., Deisseroth, K., Tsien, R.W., and Reuter, H. (1999). Calmodulin supports both inactivation and facilitation of L-type calcium channels. Nature *399*, 159–162.
28. Moreau, B., Straube, S., Fisher, R.J., Putney, J.W., Jr., and Parekh, A.B. (2005). Ca2+-calmodulin-dependent facilitation and Ca2+ inactivation of Ca2+ release-activated Ca2+ channels. J. Biol. Chem. *280*, 8776–8783.
29. Singaravelu, K., Nelson, C., Bakowski, D., de Brito, O.M., Ng, S.W., Di Capite, J., Powell, T., Scorrano, L., and Parekh, A.B. (2011). Mitofusin 2 regulates STIM1 migration from the Ca2+ store to the plasma membrane in cells with depolarized mitochondria. J. Biol. Chem. *286*, 12189–12201.
30. Peterson, B.Z., DeMaria, C.D., Adelman, J.P., and Yue, D.T. (1999). Calmodulin is the Ca2+ sensor for Ca2+ -dependent inactivation of L-type calcium channels. Neuron *22*, 549–558.
31. Gwack, Y., Srikanth, S., Feske, S., Cruz-Guilloty, F., Oh-hora, M., Neems, D.S., Hogan, P.G., and Rao, A. (2007). Biochemical and functional characterization of Orai proteins. J. Biol. Chem. *282*, 16232–16243.
32. Lis, A., Peinelt, C., Beck, A., Parvez, S., Monteilh-Zoller, M., Fleig, A., and Penner, R. (2007). CRACM1, CRACM2, and CRACM3 are store-operated Ca2+ channels with distinct functional properties. Curr. Biol. *17*, 794–800.
33. Shuttleworth, T.J. (2012). Orai3—the 'exceptional' Orai? J. Physiol. *590*, 241–257.
34. Motiani, R.K., Abdullaev, I.F., and Trebak, M. (2010). A novel native store-operated calcium channel encoded by Orai3: selective requirement of Orai3 versus Orai1 in estrogen receptor-positive versus estrogen receptor-negative breast cancer cells. J. Biol. Chem. *285*, 19173–19183.
35. Schindl, R., Bergsmann, J., Frischauf, I., Derler, I., Fahrner, M., Muik, M., Fritsch, R., Groschner, K., and Romanin, C. (2008). 2-aminoethoxydiphenyl borate alters selectivity of Orai3 channels by increasing their pore size. J. Biol. Chem. *283*, 20261–20267.
36. Zhang, S.L., Kozak, J.A., Jiang, W., Yeromin, A.V., Chen, J., Yu, Y., Penna, A., Shen, W., Chi, V., and Cahalan, M.D. (2008). Store-dependent and -independent modes regulating Ca2+ release-activated Ca2+ channel activity of human Orai1 and Orai3. J. Biol. Chem. *283*, 17662–17671.
37. Yamashita, M., Somasundaram, A., and Prakriya, M. (2011). Competitive modulation of Ca2+ release-activated Ca2+ channel gating by STIM1 and 2-aminoethyldiphenyl borate. J. Biol. Chem. *286*, 9429–9442.
38. Peinelt, C., Lis, A., Beck, A., Fleig, A., and Penner, R. (2008). 2-Aminoethoxydiphenyl borate directly facilitates and indirectly inhibits STIM1-dependent gating of CRAC channels. J. Physiol. *586*, 3061–3073.
39. Bergsmann, J., Derler, I., Muik, M., Frischauf, I., Fahrner, M., Pollheimer, P., Schwarzinger, C., Gruber, H.J., Groschner, K., and Romanin, C. (2011). Molecular determinants within N terminus of Orai3 protein that control channel activation and gating. J. Biol. Chem. *286*, 31565–31575.
40. Lis, A., Zierler, S., Peinelt, C., Fleig, A., and Penner, R. (2010). A single lysine in the N-terminal region of store-operated channels is critical for STIM1-mediated gating. J. Gen. Physiol. *136*, 673–686.
41. Ben Johny, M., Yang, P.S., Bazzazi, H., and Yue, D.T. (2013). Dynamic switching of calmodulin interactions underlies Ca2+ regulation of CaV1.3 channels. Nat. Commun. *4*, 1717.
42. Oliveria, S.F., Dell'Acqua, M.L., and Sather, W.A. (2007). AKAP79/150 anchoring of calcineurin controls neuronal L-type Ca2+ channel activity and nuclear signaling. Neuron *55*, 261–275.
43. Sharma, S., Findlay, G.M., Bandukwala, H.S., Oberdoerffer, S., Baust, B., Li, Z., Schmidt, V., Hogan, P.G., Sacks, D.B., and Rao, A. (2011). Dephosphorylation of the nuclear factor of activated T cells (NFAT) transcription factor is regulated by an RNA-protein scaffold complex. Proc. Natl. Acad. Sci. USA *108*, 11381–11386.

Current Biology, Volume *24*

Supplemental Information

Dynamic Assembly of a Membrane Signaling Complex Enables Selective Activation of NFAT by Orai1

Pulak Kar, Krishna Samanta, Holger Kramer, Otto Morris, Daniel Bakowski, and Anant B. Parekh

Supplemental Figure 1

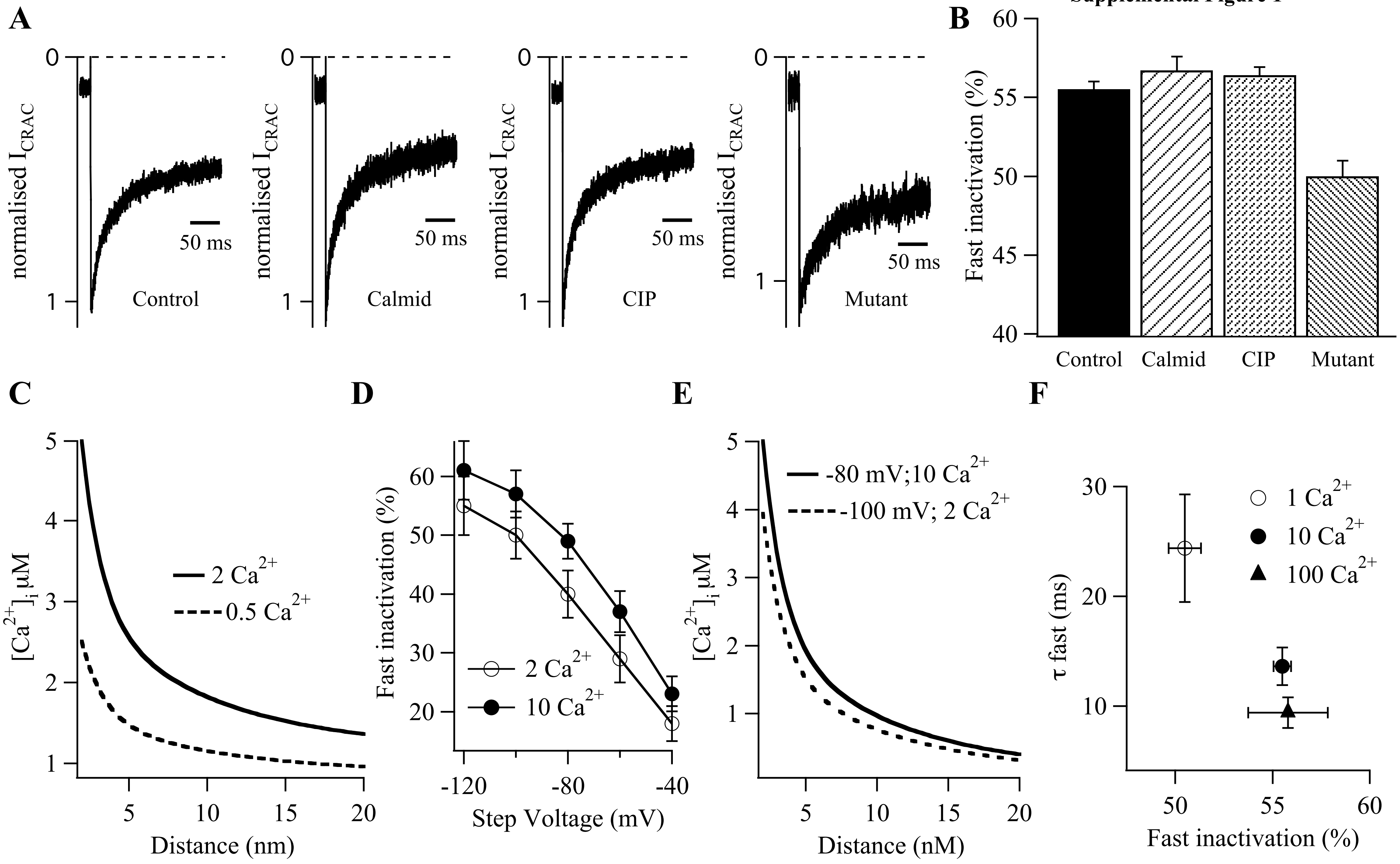

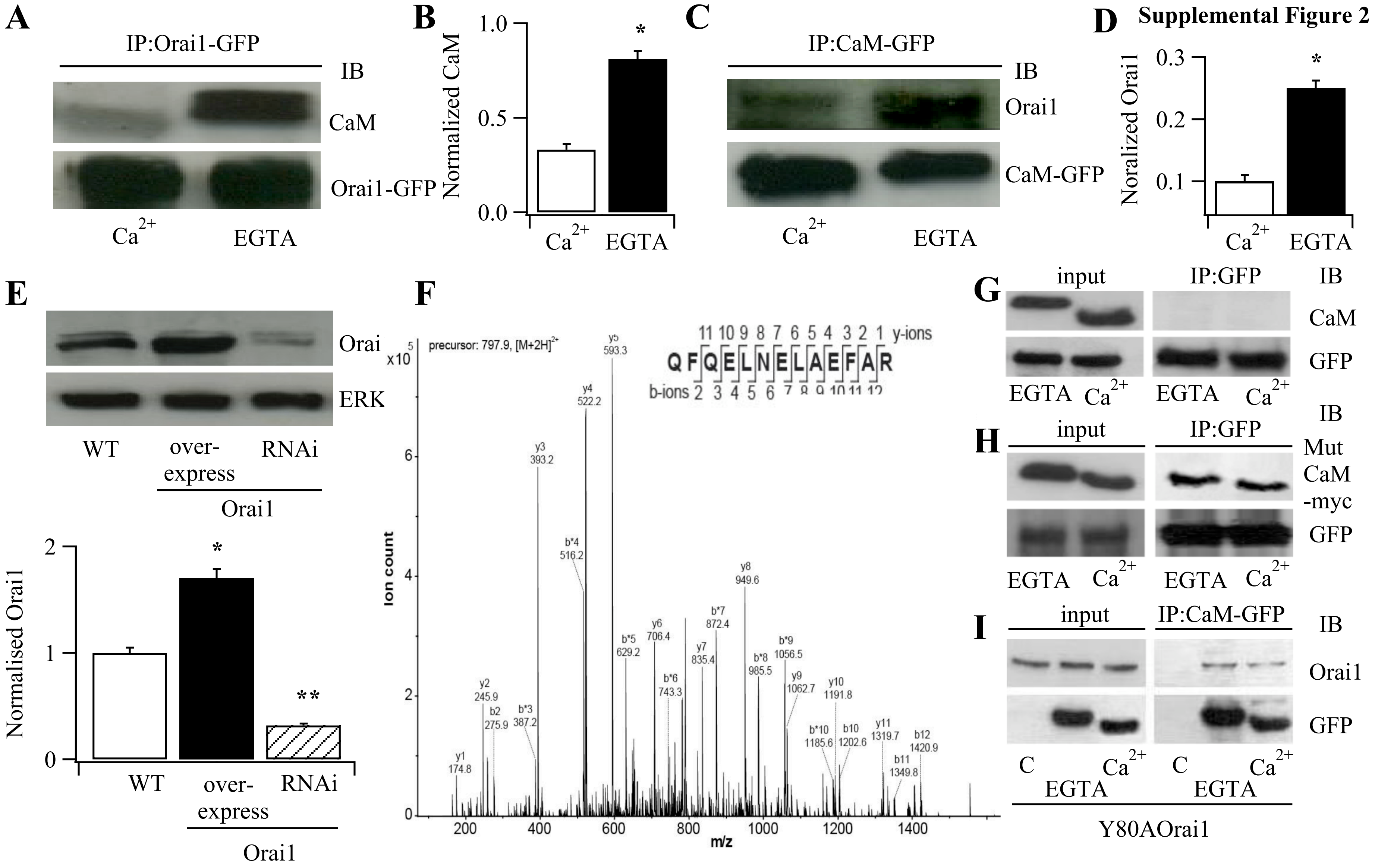
Supplemental Figure 2
A
IP:Orai1-GFP
IB
CaM
Orai1-GFP
Ca2+
EGTA
B
Normalized CaM
1.0
0.5
0.0
*
Ca2+
EGTA
C
IP:CaM-GFP
IB
Orai1
CaM-GFP
Ca2+
EGTA
D
Noralized Orai1
0.3
0.2
0.1
*
Ca2+
EGTA
E
Orai
ERK
WT
over-
express
RNAi
Orai1
Normalised Orai1
2
1
0
*
**
WT
over-
express
RNAi
Orai1
F
precursor: 797.9, [M+2H]2+
x10 5
QFQELNELAEFAR
11 10 9 8 7 6 5 4 3 2 1 y-ions
b-ions 2 3 4 5 6 7 8 9 10 11 12
Ion count
m/z
G
input
IP:GFP
IB
CaM
GFP
EGTA
Ca2+
EGTA
Ca2+
H
input
IP:GFP
IB
Mut
CaM
-myc
GFP
EGTA
Ca2+
EGTA
Ca2+
I
input
IP:CaM-GFP
IB
Orai1
GFP
C
EGTA
Ca2+
C
EGTA
Ca2+
Y80AOrai1

Supplemental Figure 3

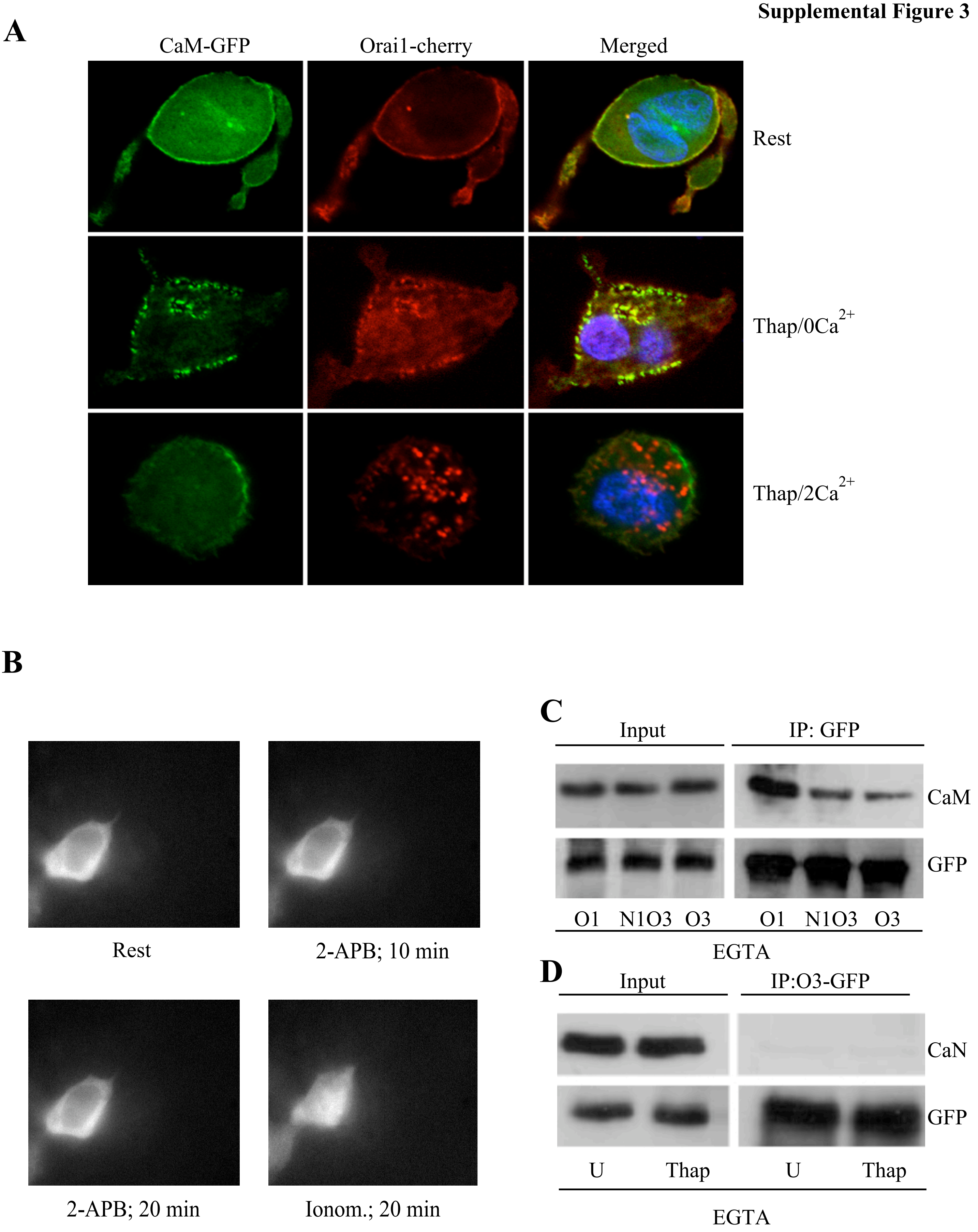

Supplemental Figure 4

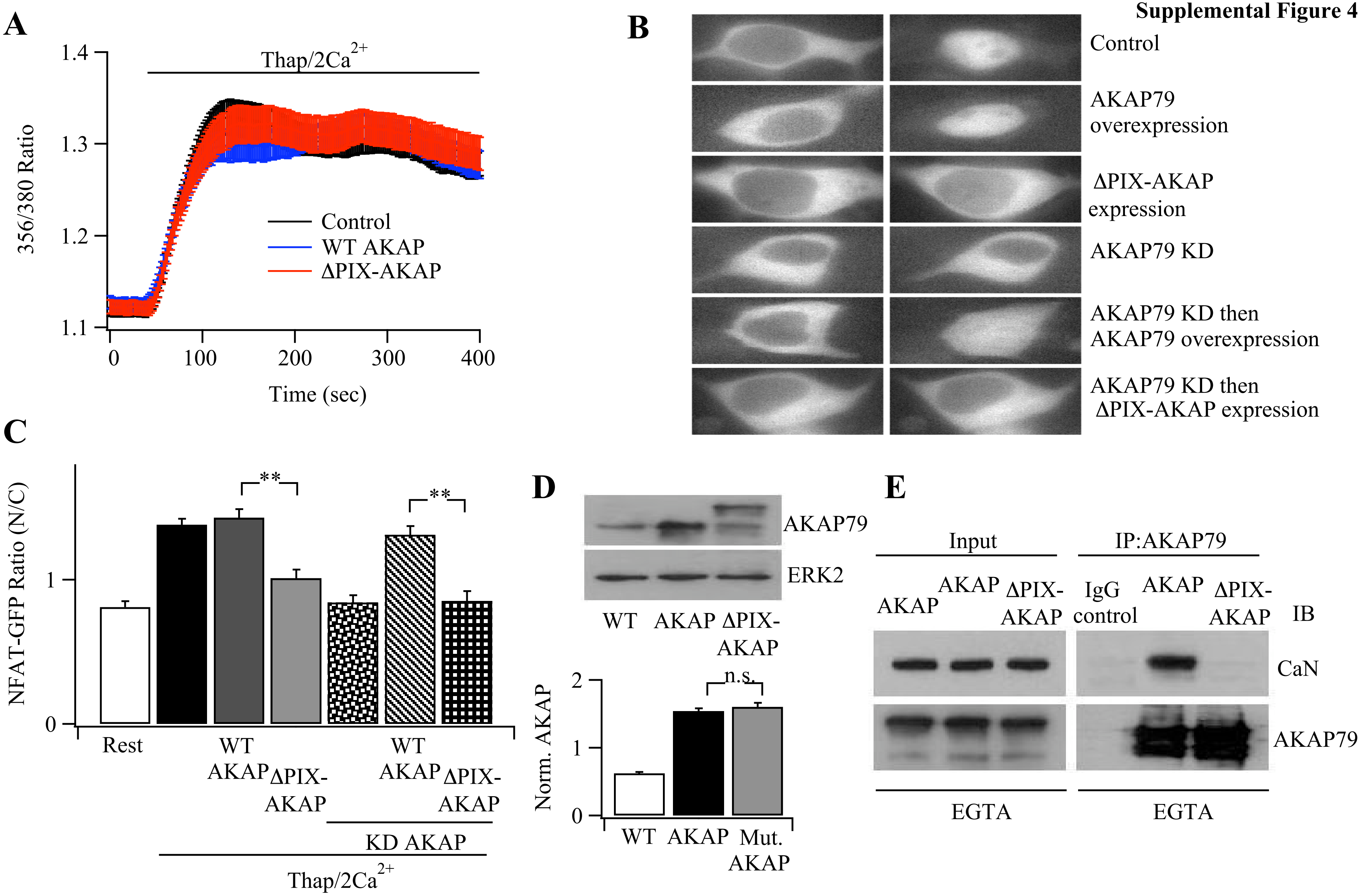

SUPPLEMENTAL

SUPPLEMENTAL FIGURE LEGEND

Supplemental Figure 1, related to Figure 2. Tethered calmodulin is close to the CRAC channel pore. A, Cells were dialysed with pipette solution containing $InsP_3$ and 10 mM EGTA and voltage steps applied for 250 ms to -100 mV from a holding potential of 0 mV. Calmid denotes calmidazolium (20 μM), CIP is calmodulin inhibitory peptide (20 μM) and mutant is a double mutant calmodulin in which Ca^{2+} binding sites in both N- and C-lobes have been lost.
Cells were pre-treated with calmidazolium for 15 minutes and the inhibitor was also added to the pipette solution. CIP was dialyzed into the cell via the pipette. For mutant, recordings were made ~ 40 hours after transfection. Cells were co-transfected with eGFP to identify transfected cells. B, Aggregate data from several experiments are compared. Each bar is the average of 8-14 cells. C, Simulation of the spatial profile of local Ca^{2+} as a function of distance from the CRAC channel pore under conditions where cytosolic Ca^{2+} is free to rise. $[Ca^{2+}]_{local} = (i_{Ca}/4*\pi*F*D_{Ca}*r) + [Ca^{2+}]_{steady\ state}$. The latter was measured (using fura 2) as ~ 1.0 μM following stimulation with thapsigargin in 2 mM Ca^{2+} and ~ 0.8 μM in 0.5 mM Ca^{2+}. Values for i_{Ca} were -2.91 fA (2 mM Ca^{2+}, estimated from noise analysis) and -1.247 fA (0.5 mM Ca^{2+}, obtained by scaling the macroscopic current to that seen in 2 mM Ca^{2+}). D_{Ca} is taken as 300 $\mu m^2/s$. r is the distance from the pore. D, Voltage-dependence of fast inactivation is compared between 2 mM and 10 mM external Ca^{2+}. CRAC channels were activated by dialysis with $InsP_3$ and 10 mM EGTA. Hyperpolarizing pulses were applied to different voltages from a holding potential of 0 mV at 0.5 Hz. E, Simulation of local Ca^{2+} following flux through CRAC channels at -80 mV in 10 mM Ca^{2+} or -100 mV in 2 mM Ca^{2+}, based on the recording conditions in panel D (high intracellular EGTA). In high

intracellular Ca^{2+} buffer, $[Ca^{2+}]_{local} = (i_{Ca}/4*\pi*F*D_{Ca}*r)*\exp(-r/\lambda)$, where λ is the mean path length of Ca^{2+} (calculated as 120 nm in 10 mM EGTA). F, Graph plots the kinetics of fast inactivation against extent of fast inactivation following CRAC channel opening under the different external Ca^{2+} concentrations shown. Each point is the average of between 7 and 13 cells.

Supplemental Figure 2, related to Figure 2. Ca^{2+}-dependent interaction between calmodulin (CaM) and Orai1. A, Following immunprecipitation of Orai-GFP on GFP-coated beads, samples were blotted for calmodulin. Lysis buffer contained either 2 mM $CaCl_2$ or 4 mM EGTA. B, Aggregate data from 4 independent experiments are compared. CaM immunoblots were normalised to Orai1-GFP levels. C, Following immunoprecipitation of calmodulin-GFP, samples were blotted for Orai1 for the two conditions shown. D, Aggregate data are summarised from 3 separate experiments. Orai1 immunoblots were normalised to CaM-GFP levels. E, The Orai1 antibody detects changes in the levels of Orai1. The histogram summarises aggregate data from 3 independent experiments. F, Identification of Orai1 by tandem mass spectrometry (LC-MS/MS). Fragmentation spectrum of a tryptic peptide leading to identification of human Orai1 (269-281) by the MASCOT algorithm with an ions score of 101; annotations: *, loss of ammonia/ deamination (-17 Da), °, loss of water/ dehydration (-18 Da). G, Following transfection with GFP alone, pulldown of GFP failed to reveal the presence of calmodulin in either EGTA or Ca^{2+}-containing lysis buffer. H, Ca^{2+}-insensitive calmodulin is associated with Orai1. Cells were transfected with Ora1-GFP and myc-tagged mutant calmodulin (an asparate residue in each of the four EF hands had been mutated to an alanine) plasmids. After pulldown of GFP, samples were blotted for myc-tagged calmodulin in lysis buffer containing either 4 mM EGTA or 2 mM Ca^{2+}. I, Y80A Orai1 binds calmodulin in the absence

(EGTA) and presence (2 mM) of Ca^{2+} in lysis buffer. C denotes control; cells that had not been transfected with CaM-GFP. These cells were then treated in the same way as those that had been transfected, and this included pulldown on GFP-coated beads. As the 'C' group had no CaM-GFP, this is a negative control.

Supplemental Figure 3, related to Figure 2 and Figure 3. 2-APB does not impair NFAT activation following a rise in Ca^{2+} evoked by a high concentration of ionomycin (5 μM). A, Confocal microscope images show distribution of calmodulin-GFP and Orai1-cherry in non-stimulated RBL cells, after store depletion with thapsigargin in Ca^{2+}-free solution and 4 minutes after readmission of external Ca^{2+} to cells treated with thapsigargin in Ca^{2+}-free solution. B, 2-APB failed to elicit NFAT-GFP movement to the nucleus in cells overexpressing Orai3. However, stimulation with ionomycin (in the presence of 2-APB) resulted in strong nuclear migration. C, Following transfection, GFP-tagged Orai1, N1-Orai3 or Orai3 were immunoprecipitated and blotted for CaM. Lysis buffer contained 4 mM EGTA. D, Following immuno-precipitation of Orai3-tagged GFP, samples were blotted for calcineurin in either untreated (non-stimulated; labelled U) cells or after store depletion following exposure to thapsigargin (2 μM) in Ca^{2+}-free solution for 5 minutes (labelled Th/0). Lysis buffer contained 4 mM EGTA.

Supplemental Figure 4, related to Figure 4. Expression of ΔPIX-AKAP79 impairs CRAC channel-dependent activation of NFAT. A, Cytoplasmic Ca^{2+} signals to thapsigargin are unaffected by overexpression of wild type AKAP79 or expression of ΔPIX-AKAP79-cherry. B, Nuclear accumulation of NFAT-GFP is compared for the different conditions. AKAPKD refers to knockdown of AKAP79. 24 hours after knockdown, cells were transfected with either wildtype AKAP79 or ΔPIX-AKAP79-cherry. C, Aggregate data from several

experiments are compared. Each bar is the mean of between 6 and 11 cells. D, Western blot compares expression of AKAP79 in wildtype cells (non-transfected), after overexpression of AKAP79 and after expression of ΔPIX-AKAP79-cherry. Note the two bands in the latter case, reflecting endogenous AKAP and tagged recombinant protein. E, Pulldown experiments show that AKAP79-YFP is associated with calcineurin in the absence of external Ca^{2+} but no interaction is seen when ΔPIX-AKAP79-CFP is expressed instead. Lysis buffer contained 4 mM EGTA. IgG control is shown, which represents pull down of AKAP79-YFP onto non-GFP beads.

SUPPLEMENTAL EXPERIMENTAL PROCEDURES

Cell Culture and Transfection-HEK293 and rat basophilic leukemia (RBL-1) cells were bought from ATCC and were cultured (37 °C, 5% CO_2) in Dulbecco's modified Eagle medium with 10% fetal bovine serum, 2 mM L-glutamine and penicillin-streptomycin, as previously described[S1]. HEK293 cells were transfected using the lipofectamine method. RBL-1 cells were transfected using the AMAXA system. RBL-1 cells were used for reporter gene expression studies (Fig. 1G, H), measurement of fast inactivation of endogenous CRAC channels (Supplementary Figure 1) and confocal microscopy of calmodulin-GFP and Orai-cherry (Supplementary Figure 3A). All other data were obtained with HEK293 cells.

cDNA Constructs-Orai1 and STIM1 were purchased from Origene. STIM1-YFP was a gift from Dr Tobias Meyer (Stanford). Orai1-GFP, Orai3-GFP and calmodulin mutant were provided by Dr. James Putney (NIEHS). NFAT-GFP was provided by Dr Paul Worley (Johns Hopkins). GFP driven by an NFAT promoter was from Dr Yuriy Usachev (Iowa). Orai1-cherry was from Dr Alexey Tepikin (Liverpool). AKAP79-YFP was kindly provided by Dr Mark Dell'Acqua (Colorado). The AKAP mutant ΔPIX variant (deletion of PIAIIIT),

which cannot bind calcineurin, was from Dr Dell'Acqua and was tagged with either CFP or cherry. The Orai1-Orai3 pentamer was kindly provided by Dr Trevor Shuttleworth (Rochester, NY). Orai1 mutants were generated by site-directed mutagenesis and verified by sequencing. A73E was initially generated using a GeneArt kit from Invitrogen and then confirmed by purchase of an A73E mutant from Mutagenex. W76A and Y80A, and the mutant myc-tagged calmodulin construct were obtained from Mutagenex.

Ca^{2+} imaging-Ca^{2+} imaging experiments were carried out at room temperature using the IMAGO CCD camera-based system from TILL Photonics, as described previously[S1]. Cells were alternately excited at 356 and 380 nm (20 msec exposures) and images were acquired every 2 seconds. Images were analysed offline using IGOR Pro for Windows. Cells were loaded with Fura 2-AM (2 μM) for 40 minutes at room temperature in the dark and then washed three times in standard external solution of composition (in mM) NaCl 145, KCl 2.8, $CaCl_2$ 2, $MgCl_2$ 2, D-glucose 10, HEPES 10, pH 7.4 with NaOH. Cells were left for 15 minutes to allow further deesterification. Ca^{2+}-free solution had the following composition (in mM) NaCl 145, KCl 2.8, $MgCl_2$ 2, D-glucose 10, HEPES 10, EGTA 0.1, pH 7.4 with NaOH). Low Na^+ external solution contained (in mM) NaCl 10, TRIS base 135, KCl 2.8, $CaCl_2$ 2, $MgCl_2$ 2, D-glucose 10, HEPES 10, EGTA 0.1, pH 7.4 with HCl).

***I_{CRAC}* recordings-**Patch-clamp experiments were conducted in the tight-seal whole-cell configuration at room temperature (20-24^{0}C) as previously described[S2]. Sylgard-coated, fire-polished pipettes had d.c. resistances of 4.2-5.5MΩ when filled with standard internal solution that contained (in mM): Cs^+ glutamate 145, NaCl 8, $MgCl_2$ 1, Mg-ATP 2, Ethylene glycol-bis(b-aminoethyl ether)-N,N,N′,N′,-tetraacetic acid (EGTA) 10, $InsP_3$ (0.03), HEPES 10, pH 7.2 with CsOH. A correction of 10 mV was applied for the subsequent liquid

junction potential that arose from this glutamate-based internal solution. The composition of the extracellular solution was (in mM): NaCl 145, KCl 2.8, $MgCl_2$ 2, CsCl 10, D-glucose 10, HEPES 10, pH 7.4 with NaOH. In some experiments, 1, 10 mM or 100 mM $CaCl_2$ was added (described in manuscript). 100 mM $CaCl_2$ solution contained 10 mM NaCl.

I_{CRAC} was measured by applying voltage ramps (-100 to +100 mV in 50 msec) at 0.5 Hz from a holding potential of 0 mV. For fast inactivation, step pulses (250 msec duration) were applied from 0 mV to -100 mV every 2 seconds. Currents were filtered using an 8-pole Bessel filter at 2.5 kHz and digitised at 100 ms. Inactivation was determined by dividing the steady state current during the hyperpolarising pulse (measured after 240 ms) by the initial current (measured after 1 ms). Capacitative currents were compensated before each ramp by using the automatic compensation of the EPC 9 -2 amplifier. Leak currents were subtracted by averaging 2-3 ramp currents obtained just before I_{CRAC} had started to develop, and then subtracting this from all subsequent currents.

TIRF microscopy-Cells were transfected with calmodulin-GFP and Orai1 24-36 hours prior to recording. TIRF recordings were carried out as described[S3].

EGTA-AM loading-Cells were loaded with EGTA by incubation for 45 minutes with EGTA-AM as described [S4].

Nuclear NFAT1-**GFP**-NFAT1-GFP levels in the cytosol and nucleus was measured using the IMAGO charge-coupled device camera-based system from TILL Photonics, with a x100 oil immersion objective (numerical aperture 1.3). Regions of interest of identical size were drawn in the cytosol and nucleus of each cell and fluorescence computed. Nuclear localization was confirmed by co-staining with a nuclear dye (DAPI), as described[S4]. Unless otherwise indicated we calculated the nuclear/cytosolic ratio of NFAT-GFP.

Gene reporter assay-24-36 hours following transfection with the EGFP-based reporter plasmid driven by an NFAT promoter, cells were stimulated with leukotriene C_4 (160 nM, 40 minutes) and the % of cells expressing EGFP measured[S4]. Gene expression was defined as fluorescence 3xSD> cell autofluorescence, measured in non-transfected cells. Cells were stimulated in culture medium and maintained in the incubator.

Co-immunoprecipitation and Western blotting-Twenty four hours after transfection, HEK293 cells were treated with 2 μM thapsigargin in Ca^{2+} free external solution for 7 minute and then lysed in 50 mM Tris-HCl (pH 7.5), 150 mM NaCl, 1% Triton X-100, and protease inhibitors with either 2 mM $CaCl_2$ (for Ca^{2+} condition) or 4 mM EGTA (for Ca^{2+} free condition) for 15 min, as described by Mullins et al. [S5]. Lysates were spun at 12000 × g for 10 min, and the supernatant was used for immunoprecipitation reaction (anti-GFP agarose beads) at 4°C. After washing four times with ice cold lysis buffer, followed by resuspension in 2X SDS sample buffer, samples were heated at 95°C for 5 min and resolved by 10% SDS–PAGE and subjected to transfer into the nitrocellulose membranes. Membranes were blocked with 5% non-fat dry milk in PBS plus 0.1% Tween 20 (PBST) buffer for 1 hour at room temperature. Membranes were washed with PBST three times and then incubated with appropriate primary antibodies for 24 hours at 4°C. Total ERK 2 and Orai-1 (Santa Cruz Biotechnology), calmodulin (Abcam), AKAP79 (BD Transduction Laboratories), Calcineurin A subunit and GFP (Cell signaling) primary antibodies were used at dilutions of 1:5000 (ERK2), 1: 1000 (orai-1, calmodulin, AKAP79, CaN and GFP). The membranes were then washed with PBST again and incubated with 1:2500 dilutions of peroxidase-linked anti-rabbit (Santa Cruz Biotechnology) or anti mouse IgG (BD Bioscience) for 1 hour at room temperature. After washing with PBST, the bands were detected by an enhanced chemiluminescence ECL-plus

Western blotting detection system (GE Healthcare). Blots were analyzed by UN-Scan IT software.

Mass spectroscopy-In-gel trypsin digestion: Gel bands of interest were excised after Coomassie blue staining and cut into 1 – 2 mm^3 gel pieces, which were placed into 1.5 mL sample tubes. Gel pieces were rinsed twice with wash solution for 18h in total (200 µL, 50% methanol, 5% acetic acid). The solutions were removed and gel pieces were dehydrated in acetonitrile (200 µL, 5 min). Supernatant were removed and gel pieces were dried in a vacuum centrifuge for 3 min. Disulfide reduction was performed with 10 mM DTT (30 µL) for 0.5 h, followed by alkylation with 100 mM iodoacetamide (30 µL) for 0.5 h. Supernatants were removed from the gel samples and dehydration with acetonitrile and evaporation performed as described above. Gel pieces were washed with 100 mM ammonium bicarbonate (200 µL, 10 min). Supernatants were removed and dehydration performed with acetonitrile and evaporation as above. The gel samples were then rehydrated on ice with freshly prepared trypsin solution (30 µL, 20 ng/µL sequencing grade trypsin [Promega] in 50 mM ammonium bicarbonate). After rehydration excess trypsin solution was removed and 50 mM ammonium bicarbonate (10 µL) was added to prevent dehydration of gel pieces. Gel samples were digested at 37ºC for 18h. The gel pieces were then extracted sequentially with 50mM ammonium bicarbonate (60 µL), 50% acetonitrile, 5% formic acid (60 µL) and 85% acetonitrile, 5% formic acid (60 µL). The combined extracts were evaporated in a vacuum centrifuge and were redissolved in 5% acetonitrile, 0.1% formic acid (20 µL) on an ultrasonic bath and transferred into LC-MS sample vials.

LC-MS/MS analysis-For the analysis of in-gel digested protein material, liquid chromatography was performed using an Ultimate 3000 nano-HPLC system (Dionex, Sunnyvale, CA, USA) comprising a WPS-3000 micro auto sampler, a FLM-3000 flow manager and

column compartment, a UVD-3000 UV detector, an LPG-3600 dual-gradient micro-pump, and an SRD-3600 solvent rack controlled by Hystar (Bruker Daltonics, Billerica, MA, USA) and DCMS link 2.0 software. Samples were concentrated on a trapping column Dionex (Sunnyvale, CA, USA), 300 µm i.d., 0.1 cm) at a flow rate of 20 µL/min. For the separation with a C18 Pepmap column (75 µm i.d., 15 cm, Dionex), a flow rate of 250 nL/min was used as generated by a cap-flow splitter cartridge (1/1000). Peptides were eluted by the application of a 30 min multi-step gradient using solvents A (98% H_2O, 2% acetonitrile, 0.1% formic acid) and B (80% acetonitrile, 20% water, 0.1% formic acid):

Composition (% solvent B)	Run time (min)
2-10	0-3
10-25	3-18
25-50	18-30
50-90	30-30.2

The liquid chromatography was interfaced directly with a 3D high capacity ion trap mass spectrometer (amaZon; Bruker Daltonics) utilizing 10 µm i.d. distal coated SilicaTips (New Objective, Woburn, MA, USA) and nano-ESI mode. SPS parameter settings on the ion trap were tuned for a target mass of 850 *m/z*, compound stability 100% and a smart ICC target of 250,000. MS/MS analysis was initiated on a contact closure signal triggered by HyStar software (version 3.2). Up to five precursor ions were selected per cycle with active exclusion (0.5 min) in collision-induced dissociation (CID) mode. CID fragmentation was achieved using helium gas and a 30%–200% collision energy sweep with amplitude 1.0 (ions are ejected from the trap as soon as they fragment).

Data processing and database searching-Raw LC-MS/MS data were processed and Mascot compatible files were created using

DataAnalysis 4.0 software (Bruker Daltonics). Database searches were performed using the Mascot algorithm (version 2.4) and the UniProt_SwissProt database with mammalian taxonomy restriction (v2012.09.17, number of entries 537,505, after taxonomy filter: 66,032). The following parameters were applied: 2+, 3+ and 4+ ions, peptide mass tolerance 0.3 Da, $^{13}C = 2$, fragment mass tolerance 0.6 Da, number of missed cleavages: two, instrument type: ESI-TRAP, fixed modifications: Carbamidomethylation (Cys), variable modifications: Oxidation (Met).

Statistics-Results are presented as means ± S.E.M. Statistical significance was assessed using Student's t test for comparison between two groups or analysis of variance (ANOVA) followed by a *post hoc* Newman Keuls multiple comparison test for the difference between groups and considered significant at $p < 0.05$ (*); $p<0.01$ (**).

SUPPLEMENTAL REFERENCES

[S1]. Kar P, Bakowski D, Di Capite J, Nelson C, & Parekh AB (2012) Different agonists recruit different stromal interaction molecule proteins to support cytoplasmic Ca2+ oscillations and gene expression. *Proceedings of the National Academy of Sciences USA* 109:6969-6974.

[S2]. Bakowski D, Glitsch MD, & Parekh AB (2001) An examination of the secretion-like coupling model for the activation of the Ca2+ release-activated Ca2+ current ICRAC in RBL-1 cells. *Journal of Physiology (Lond.)* 532:55-71.

[S3]. Singaravelu K*, et al.* (2011) Mitofusin 2 regulates STIM1 migration from the Ca2+ store to the plasma membrane in cells with depolarised mitochondria. *Journal of Biological Chemistry* 286:12189-12201.

[S4]. Kar P, Nelson C, & Parekh AB (2011) Selective activation of the transcription factor NFAT1 by calcium microdomains near Ca2+ release-activated Ca2+ (CRAC) channels. *Journal of Biological Chemistry* 286:14795-14803.

[S5]. Mullins FM, Park CY, Dolmetsch RE, & Lewis RS (2009) STIM1 and calmodulin interact with Orai1 to induce Ca2+-dependent inactivation of CRAC channels. *Proceedings of the National Academy of Sciences USA* 106:15495-15500.
